# Spatially patterned stiffness variation in a light-triggered jumper for symmetry breaking and high snap-through efficiency

**DOI:** 10.1126/sciadv.adx8301

**Published:** 2025-08-29

**Authors:** Min Jeong Hahm, Woongbi Cho, Jisoo Jeon, Hak-Rin Kim, Teng Zhang, Jeong Jae Wie

**Affiliations:** ^1^ Department of Organic and Nano Engineering, Hanyang University, 222 Wangsimni-ro, Seongdong-gu, Seoul 04763, Republic of Korea.; ^2^ Human-Tech Convergence Program, Hanyang University, 222 Wangsimni-ro, Seongdong-gu, Seoul 04763, Republic of Korea.; ^3^ School of Materials Science and Engineering, Georgia Institute of Technology, Atlanta, GA 30332, USA.; ^4^ School of Electronic and Electrical Engineering, Kyungpook National University, 80 Daehak-ro, Buk-gu, Daegu 41566, Republic of Korea.; ^5^ School of Electronics Engineering, Kyungpook National University, 80 Daehak-ro, Buk-gu, Daegu 41566, Republic of Korea.; ^6^ Department of Mechanical and Aerospace Engineering, Syracuse University, Syracuse, NY 13244, USA.; ^7^ BioInspired Syracuse: Institute for Material and Living Systems, Syracuse University, Syracuse, NY 13244, USA.; ^8^ Department of Chemical Engineering, Hanyang University, 222 Wangsimni-ro, Seongdong-gu, Seoul 04763, Republic of Korea.; ^9^ The Research Institute of Industrial Science, Hanyang University, 222 Wangsimni-ro, Seongdong-gu, Seoul 04763, Republic of Korea.; ^10^ Department of Chemical Engineering, State University of New York College of Environmental Science and Forestry, Syracuse, NY 13210, USA.; ^11^ The Michael M. Szwarc Polymer Research Institute, State University of New York College of Environmental Science and Forestry, Syracuse, NY 13210, USA.

## Abstract

The nonlinear strain response of soft material–based snap-through systems enables amplified and accelerated force output. However, efficiency of snap-through energy release is challenging to improve because of the inherent trade-off between initial curvature and stiffness. Here, spatial programming of stiffness variation in the azobenzene-functionalized liquid-crystalline polymer (Azo-LCP) addresses this limitation and achieves efficient photomechanical jumping. Introduction of stiffness mismatch induced localized curvature, which preserved the initial curvature and simultaneously enhanced photomechanical strain responsivity. By programming for symmetry of stiffness variation, we achieved directional or vertical jumping via strategic placement of the rigid region, with corresponding stress accumulation behaviors corroborated by finite element simulations. Integration of patterned stiffness variation with geometric asymmetry enabled both vertical and horizontal jumping within a single structure, without compromising performance. This dual-mode jumper also demonstrated sequential and consecutive jumps under continuous light exposure.

## Teaser

Patterned stiffness variation and geometric asymmetry in liquid crystalline polymer networks enhance the snap-through.

## INTRODUCTION

Snap-through instability is an efficient strategy for achieving rapid and powerful actuation through nonlinear energy storage and release mechanisms in small living organisms (*1*–*3*). For example, snap-through instability enables the Venus flytrap (*Dionaea muscipula*), a carnivorous plant, to capture insects within ~100-ms time span, notably the fastest movement recorded in the plant kingdom. A Venus flytrap achieves this feat despite its body being primarily composed of soft and flexible plant cells (*3*). To mimic such natural phenomena, snap-through instability has widely been explored as a power amplification strategy in artificial soft actuators and robotics (*4*–*9*). This mechanism enables soft and limbless systems to overcome their inherent limitations of softness and low actuation speeds to perform powerful jumps. Unlike the linear stress-strain relationship governed by Hooke’s law (i.e., σ = *E* × ε, where σ and ε represent applied stress and strain, respectively, and *E* is the elastic modulus), energy accumulation and release mechanisms of snap-through systems are characterized by a nonlinear topological transition between distinct nonisometric equilibrium states (fig. S1) (*10*, *11*). This phenomenon is commonly referred to as bistability. When an elastic strip has fixed ends, such that the length is shorter than that in its original state, the film initially adopts a stable curved form with a single Gaussian curvature at equilibrium. Then, as the apex is compressed with an external force, the strip deforms into an energetically unstable intermediate state. Once the applied force exceeds the energy barrier for snap-through, the strip rapidly transitions into an inversely curved equilibrium configuration and releases the accumulated energy as kinetic energy.

Snap-through behavior arises from the interplay of material and geometric properties in systems with unconstrained ends (*4*, *9*). An analogous shell-like structure was adopted by Jeon *et al.* (*9*) to demonstrate snap-through–based jumping of a system based on an azobenzene-functionalized liquid-crystalline polymer (Azo-LCP). Azo-LCP is an intelligent organic material that features a cross-linked network structure through covalent bonding between liquid crystal (LC) molecules and ultraviolet (UV)–responsive azobenzene moieties. The Azo-LCP reconfigures its shape in response to temperature or UV light, guided by the programmed molecular alignment of LC molecules (*12*–*16*). UV light–triggered *trans*-*cis* isomerization of the azobenzene moiety induces photomechanical strain that triggers a snap-through accompanied by a rapid release of the accumulated stress (fig. S2). However, the snap-through–driven jumping performance is fundamentally constrained by a trade-off relationship between initial curvature and mechanical stiffness, both of which critically affect the snap-through energy barrier. Jumping performance of a snap-through system is directly proportional to the snap-through energy barrier, which adheres to the action-reaction principle. Moreover, the magnitude of the snap-through energy barrier increases proportionally with both initial curvature and mechanical stiffness of the system (*17*–*19*). While a higher initial curvature raises the critical strain for snap-through, a larger elastic modulus increases the amount of elastic energy stored per unit strain (*20*–*22*). Ideally, a system combining high initial curvature and large modulus can achieve a higher energy barrier for an enhanced snap-through energy release. However, a trade-off exists between the initial curvature and mechanical stiffness of unconstrained systems, and Hooke’s law demands that a modulus increase leads to a decreased initial curvature under similar levels of external forces. As a result, the amount of energy released from snap-through typically peaks at an intermediate balance, making it a challenge to achieve synergistic enhancement through simultaneous engineering of both parameters. This fundamental trade-off highlights the need for a new design strategy that enables simultaneous realization of high initial curvature and high mechanical stiffness by spatially programming stiffness variation and incorporating asymmetric geometry within a single structure.

Here, we spatially patterned for mechanical stiffness variation of Azo-LCP to overcome the inherent trade-off between material stiffness and initial curvature. To introduce heterogeneity in stiffness, we spatially controlled cross-linking density of the Azo-LCP monolithic film by locally varying photopolymerization time. The patterned stiffness variation design integrates high photomechanical strain and high mechanical stiffness to extend the energy storage pathway and increase stress accumulation efficiency, resulting in a synergistic elevation of the snap-through energy barrier. We implemented two key design strategies: (i) asymmetric positioning of the rigid region at the corner and (ii) symmetric positioning of the rigid region at the center. The corner- and center-rigid Azo-LCPs enabled highly efficient directional jumps and vertical jumps, respectively, under identical UV light irradiation. A corner-rigid design caused tilted energy release during sequential snap-through from rigid to soft region, which generated rotational torque and directional jumps. In contrast, the center-rigid design achieved a record-high jump height by combining high initial curvature at the soft edges with effective stress accumulation at the rigid center. Last, we demonstrated dual-mode jumping in a single Azo-LCP by integrating a soft-rigid alternating pattern with an asymmetric geometry (aspect ratio of >1). Because of the orthogonal alignment of LC molecules on opposing surfaces, each side exhibited a distinct photomechanical shape reconfiguration. UV light applied to the surface with LC alignment parallel to the long axis induced an end-to-end overlap, generating a blocking force–assisted snap-through and resulting in a high vertical jump. Conversely, UV irradiation on the surface with LC alignment along the short axis, in combination with the internal stiffness gradient, triggered a biased snap-through that led to a directional jump. Notably, the dual-mode Azo-LCP jumper has nearly twice the mass of its single-mode counterparts, yet it achieves comparable jump height and distance, highlighting the efficiency of this integrated design. This work presents a unified platform that combines stiffness variation and geometric asymmetry to maximize energy-conversion efficiency and control force directionality in untethered soft actuators driven by snap-through.

## RESULTS

### Spatial programming of stiffness variation in Azo-LCPs

**Fig. 1.**
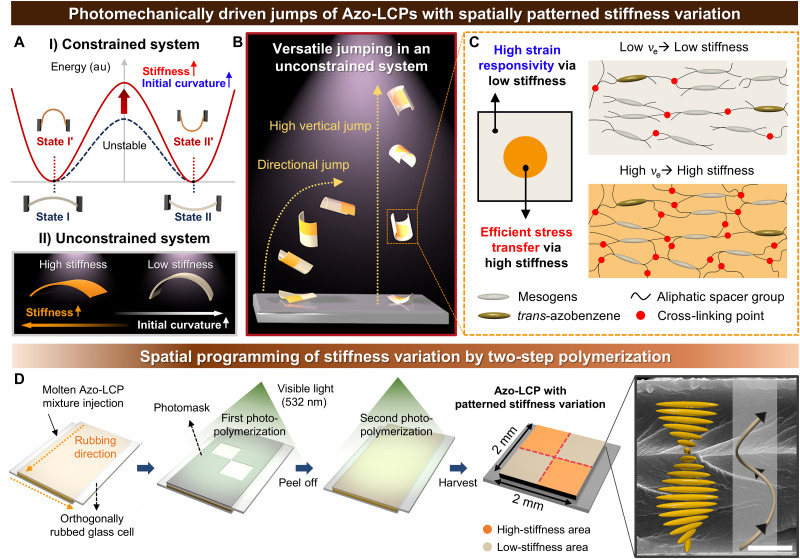
Spatial programming of stiffness variation in Azo-LCP. (**A**) Schematic illustration of relationship between stiffness and initial curvature on snap-through energy barrier of (I) a constrained system and (II) an unconstrained system. au, atomic unit. (**B**) Three-dimensional (3D) illustration of photomechanical jumps of Azo-LCPs with patterned mechanical stiffness variation. (**C**) Schematic illustration of patterned Azo-LCP film regions of high and low stiffness based on cross-linking density (ν_e_) differences. (**D**) Scheme of Azo-LCP film preparation with stiffness variation using masking techniques, along with a 3D schematic illustration and an actual scanning electron micrograph capturing 270° supertwisted nematic (STN) alignment of Azo-LCP. Scale bar, 5 μm.

We pursued a design approach that would enable versatile jumping of an Azo-LCP film via strain engineering while simultaneously overcoming the trade-off between strain responsivity and energy release efficiency of the snap-through system (Fig. 1, A and B). To achieve this, we spatially patterned mechanical stiffness variation of the Azo-LCP film, forming defined regions of higher and lower mechanical stiffness within a single structure. As illustrated in Fig. 1C, for example, different strain responses were encoded into the structure by varying cross-linking density across distinct sections of Azo-LCP. The cross-linking density of Azo-LCP was controlled by photopolymerizing the LC monomer with spatiotemporal control of photoirradiation, as prolonged photocuring leads to a greater cross-linking density, thus inducing formation of additional cross-links between LCP chains. Loosely cross-linked regions on the photopatterned Azo-LCP film exhibited a high photomechanical strain response owing to their low elastic modulus, enabling a greater initial curvature. Conversely, highly cross-linked regions effectively accumulated stress, even under the same photogenerated strain, due to their higher elastic modulus. By strategically implementing these regions in a tailored pattern, the advantage of each could be harnessed, efficiently enhancing jumping performance of the system under identical external stimuli.

A two-step photopolymerization process, including a masking process, was used to produce Azo-LCPs with patterned stiffness (Fig. 1D). The Azo-LCP precursor consisted of a reactive mesogen, an acrylate-functionalized azobenzene serving as a photoactive molecular switch, a chiral dopant, and a photoinitiator (fig. S3). The molten Azo-LCP precursor, heated to a temperature above nematic-to-isotropic temperature (*T*_NI_), was injected into a glass cell prepared by attaching two mechanically rubbed glass slides with linear surface relief grating oriented orthogonally to each other. Then, a photomask was attached to the glass substrate, and the Azo-LCP mixture was cooled to achieve nematic phase. Subsequently, LC molecules were self-assembled along the orthogonal alignment of surface relief gratings on the top and bottom glass slides. The helical twisting power of the chiral dopant facilitated the formation of 270° supertwisted nematic (STN) directors along the thickness direction. In this first step of photopolymerization, uncovered regions were polymerized with green light (532 nm) at an intensity of ~0.05 W cm^−2^. Then, the photomask was removed, and the entire Azo-LCP sample was cured to achieve the desired cross-linking density difference. A 270° STN alignment was confirmed via cross-sectional scanning electron micrograph; the middle layer, featuring a 180° rotation in molecular alignment, acted as a stress-neutralization area, reducing through-thickness strain gradient and suppressing excessive bending that would otherwise lead to jump failure.

### Thermomechanical properties of uniformly cross-linked Azo-LCPs

**Fig. 2.**
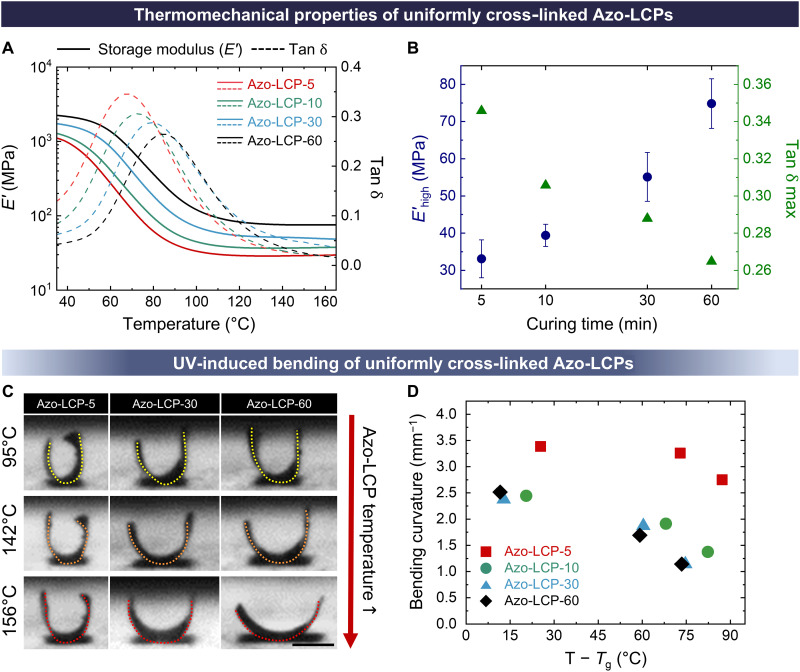
Thermomechanical properties and UV-induced deformation of uniformly cross-linked Azo-LCPs. (**A**) Thermomechanical properties evaluated by DMA. Temperature-dependent storage modulus (*E*′; solid line) and tan δ (dashed line) of Azo-LCPs cured for 5, 10, 30, and 60 min are displayed using red, green, blue, and black lines, respectively. (**B**) Storage modulus at rubbery plateau region (*E*′_high_) and peak of tan δ plotted against curing time of Azo-LCPs. (**C**) Photographs showing bending deformation of Azo-LCPs under UV irradiation at 0.3 W cm^−2^ intensity. Scale bar, 1 mm. (**D**) Measured UV-induced bending curvatures depending on Azo-LCP temperature.

To confirm the effect of photopolymerization time on cross-link density and mechanical stiffness, we analyzed the thermomechanical properties of Azo-LCPs cured uniformly for different durations (5, 10, 30, and 60 min). The measured storage modulus (*E*′), loss modulus (*E*″), and tan δ (= *E*″/*E*′) values of cured Azo-LCPs were plotted as a function of temperature (Fig. 2A and fig. S4). Samples are referred to as Azo-LCP-XX, where XX indicates photopolymerization time in minutes. Glass-transition temperature (*T*_g_) was determined from peak temperature of tan δ plot obtained through dynamic mechanical analysis (DMA). A shorter polymerization time increased free volume and conformational entropy of polymer chains between cross-links, and measured *T*_g_ decreased from 85.4° to 67.4°C. On the other hand, the measured tan δ increased from 0.04 to 0.12 with a decrease in photopolymerization time, implying enhanced damping properties due to the dangling chains. When temperature was elevated above *T*_g_, the modulus decreased substantially for all Azo-LCPs, and rubbery plateau regions indicated successful formation of network structures. Furthermore, storage modulus at the rubbery plateau region (*E*′_high_) decreased from 74.8 to 33.1 MPa as photopolymerization time was reduced from 60 to 5 min. The *E*′_high_ and tan δ peak are summarized in Fig. 2B. These data were then correlated with cross-linking density (ν_e_) using Flory’s rubber elasticity theory described in Eq. 1 (*23*)

νe=Ehigh′/3RThigh (1)

where *E*′_high_ is the storage modulus at *T*_high_, the temperature in the rubbery plateau region that typically corresponds to *T*_g_ + 50°C, and *R* is the ideal gas constant. Reducing photopolymerization time effectively lowered the cross-linking density from 7388 to 3382 mol m^−3^, and this clear decreasing trend is listed in fig. S5A and table S1. A decreased cross-linking density resulted in a softer and more ductile Azo-LCP, as indicated by a reduction in modulus from 18.89 to 6.82 MPa and tensile strength from 16.43 to 15.02 MPa, along with an increased elongation at break from 1.34 to 6.75% (fig. S5B and table S2).

Varied mechanical stiffness induced distinct shape reconfigurability upon top-down UV light irradiation at 0.3 W cm^−2^ intensity, as shown in Fig. 2C. Upon UV exposure, Azo-LCP bent toward the UV-exposed side via skin-bulk effect. Exponential decay of the UV intensity, as dictated by Beer-Lambert law, activated azobenzene units near the surface, which then underwent *trans*-*cis* photoisomerization to generate stress and trigger an LC order-disorder transition. Azo-LCPs with dimensions of 2 mm by 2 mm were subjected to different body temperatures, and the measured negative bending curvatures (*R*^−1^) are plotted in Fig. 2D. As a result, Azo-LCP-5 showed a maximum curvature of 3.4 mm^−1^, whereas other Azo-LCPs with higher cross-linking density exhibited a maximum curvature of ~2.5 mm^−1^. Loosely cross-linked Azo-LCP-5 exhibited higher conformational entropy due to the longer chain length between cross-linking points. Consequently, Azo-LCP-5 showed notably larger and more responsive UV-induced bending deformation compared to more densely cross-linked Azo-LCPs, despite having the same azobenzene content, owing to its enhanced photomechanical strain responsivity. In addition, the curvature decreased with increasing temperature, as enhanced effect of thermally induced *cis*-*trans* back isomerization reduced photogenerated stress. At lower temperatures, thermal back isomerization of *cis*-azobenzene is suppressed because the thermal energy is insufficient to overcome the activation barrier for π-π* transition of the N═N double bond. Conversely, temperature elevation provides greater thermal energy to the molecules, facilitating back isomerization to the more energetically stable *trans* configuration. Compared to Azo-LCP-60, Azo-LCP-5 exhibited a smaller decrease in bending curvature at elevated temperatures, owing to its larger and more responsive photogenerated strain. Specifically, Azo-LCP-5 showed only a slight reduction from 3.4 to 2.8 mm^−1^, whereas Azo-LCP-60 experienced a more pronounced decrease from 2.5 to 1.1 mm^−1^.

### Stiffness-dependent snap-through and jumping performance

**Fig. 3.**
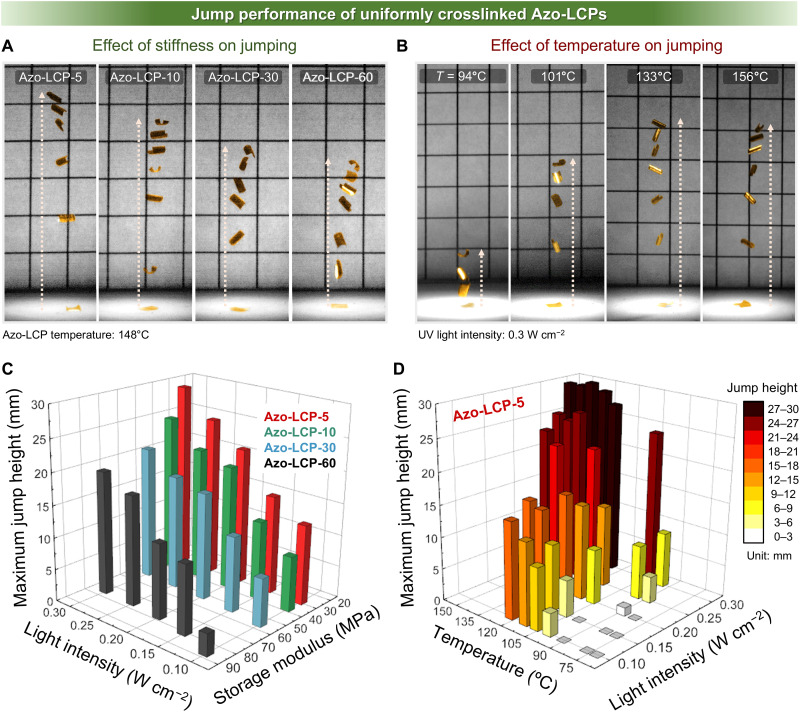
Effects of mechanical stiffness and body temperature on photomechanical jumping performance of Azo-LCPs. Digital images illustrate vertical photomechanical jumping of (**A**) Azo-LCPs with varying cross-linking density under a constant UV light intensity, and (**B**) Azo-LCP-5 at different body temperatures under a constant UV light intensity. Grid, 5 mm. (**C**) Jump heights of Azo-LCPs with varying cross-linking densities under various light intensities. (**D**) Jump heights of Azo-LCP-5 at different body temperatures and under different light intensities.

The mechanical stiffness- and temperature-dependent jump heights of uniformly cross-linked Azo-LCPs were systematically investigated (Fig. 3, A and B, and movies S1 and S2). Among Azo-LCPs with varied mechanical stiffness, Azo-LCP-5 achieved the greatest jump height under all tested conditions (Fig. 3C and figs. S6 and S7). Furthermore, jump height gradually increased as mechanical stiffness was reduced. Jumping in Azo-LCPs is initiated through conversion of light energy into elastic energy, which accumulates during the process of deformation from a monoclastic (+) to an anticlastic structure over the critical time for snap-through (*t*_cr_). Once stored elastic energy exceeds the snap-through energy barrier, defined by critical strain reached at *t*_cr_, the anticlastic structure rapidly transitions into a monoclastic (−) geometry. At this moment, a photomechanical jump is triggered if the released energy is sufficient to overcome gravitational potential energy. According to the principle of energy conservation, the observed jump height reflects the magnitude of the snap-through energy barrier. Therefore, the highest jump height of Azo-LCP-5 indicates the highest energy barrier compared to other Azo-LCPs with greater mechanical stiffness.

Conventional constrained systems, such as clamped strip-type geometries, allow artificial induction of large initial curvature regardless of stiffness. In contrast, our Azo-LCP jumper has fixed dimensions and unconstrained edges, where initial curvature develops intrinsically from material response. In this unconfined configuration, higher mechanical stiffness naturally leads to smaller initial curvature and reduced strain response under identical UV irradiation conditions, thereby limiting achievable snap-through energy barrier (fig. S8). As shown in fig. S9A, Azo-LCP-60 exhibited a lower initial curvature than Azo-LCP-5 during UV-induced deformation from monoclastic (+) to anticlastic structure. An increase in initial curvature leads to a greater reduction in the projected length (*L*) between the two edges compared to the original length (*L*_0_ = 2 mm), and this reduction in *L*/*L*_0_ increases the critical strain and energy barrier required for snap-through (*10*, *19*). Furthermore, Azo-LCP-60 showed consistently longer *t*_cr_ than Azo-LCP-5 across all tested light intensities from 0.02 to 0.3 W cm^−2^, indicating a reduced photomechanical strain rate associated with higher stiffness (fig. S9B)_._ The slow photomechanical strain response greatly deteriorates conversion ratio from snap-through to jumping. As shown in fig. S10A, Azo-LCPs exhibit snap-through behavior even under the low UV light intensity of 0.02 W cm^−2^, but this snap-through does not lead to a jump due to the markedly prolonged *t*_cr_. While both long exposure to low-intensity UV and short exposure to high-intensity UV provide similar energy input to induce snap-through, their effectiveness in storing elastic energy differs substantially due to the viscoelasticity of Azo-LCPs. Under prolonged low-intensity exposure, slow accumulation of photogenerated strain provides sufficient time for LC networks to undergo segmental and translational motions. This molecular-level stress relaxation increases energy dissipation and decreases the net accumulated elastic energy. In contrast, high-intensity UV exposure over a short duration induces rapid photomechanical deformation, which minimizes opportunity for molecular relaxation. As a result, elastic energy is stored more efficiently, and the initial curvature is better preserved, allowing the structure to more effectively maintain a high snap-through energy barrier and achieve higher jumping performance. Although Azo-LCP-60 enables more efficient stress accumulation per unit strain due to its higher stiffness, its lower initial curvature and longer *t*_cr_ limit the enhancement of jumping performance. Consequently, Azo-LCP-60 requires a higher threshold of UV light intensity for jumping compared to Azo-LCP-5 (fig. S10B). Furthermore, Azo-LCP-60 consistently exhibited lower jumping performance than Azo-LCP-5 across all tested conditions (fig. S10, C and D).

The combination of higher initial curvature and faster photomechanical strain response enabled Azo-LCP-5 to achieve greater jump heights than those of mechanically stiffer Azo-LCPs. Notably, Azo-LCP-5 achieved a maximum height of 29.1 mm, which is 14.6 times its body length (BL) and 1455 times its thickness (Fig. 3D and figs. S11 and S12). Increasing light intensity resulted in a larger jump height via photodynamically enhanced *trans*-*cis* isomerization, driven by the accelerated excitation rate of azobenzene moieties (*24*). In addition, the intensified light populated higher excited states that decay to the first excited state where isomerization occurs (*24*). With a faster isomerization rate, Azo-LCP facilitates deformation into an anticlastic structure, contributing to an improved jumping performance. Elevated temperature increased the rate of *cis*-*trans* back isomerization, which reduced UV penetration depth and amplified strain mismatch across film thickness (*9*). This led to a greater initial curvature and stress accumulation via skin-bulk effect, thereby enhancing jump height. However, above ~140°C, excessive back isomerization limited photogenerated strain buildup, resulting in saturation of jumping performance. Jumping performance of Azo-LCP-5 was also evaluated on substrates with varied surface roughness, and despite minor variations in magnitude, the overall trends were consistently maintained (fig. S13). Moreover, lower *T*_g_ of Azo-LCP-5 also enhanced its jumping performance under milder actuating conditions as the rubbery plateau region appeared earlier and reduced the temperature at which a substantial decrease in modulus occurs. Consequently, increased initial curvature and strain responsivity were achieved at lower energy inputs. Through mechanical-stiffness engineering, Azo-LCP-5 jumping was initiated at ~100°C, 0.1 W cm^−2^ and 90°C, 0.2 W cm^−2^, achieving a maximum jump height of 16 and 25 mm, respectively. This jump height is remarkably higher than that reported for the previous Azo-LCP–based jumping system, which exhibited the first jump at ~120° and ~150°C with a maximum jump height of 7 and 16 mm, respectively, under identical UV irradiation conditions (fig. S14) (*9*). However, the inherent ductility of Azo-LCP-5 poses a fundamental trade-off: The amount of stress accumulated per unit strain is lower than that of stiffer samples, limiting its capabilities.

### Breaking the symmetry via asymmetrically patterned stiffness variation

Symmetry breaking is one of the strategies adopted for introducing directionality into motions. Our patterning strategy enables facile symmetry breaking through asymmetric pattern adaptations, resulting in tilted energy release. We induced asymmetrically patterned stiffness variation by integrating soft and rigid areas within a single LCP body of fixed dimensions. Initially, photopolymerization times were varied to obtain soft and rigid regions to systematically evaluate jumping performance while maintaining equal ratios of the two types of areas. Jump height gradually increased as stiffness disparity between soft and rigid regions increased (fig. S15). Moreover, a greater stiffness disparity between soft and rigid regions enabled the rigid domain to better retain its initial curvature during topological transition, inducing higher geometrical asymmetry (fig. S16). On the basis of these observations, we adopted the 5- to 60-min curing combination for soft and rigid regions, respectively, in all subsequent patterning procedures.

**Fig. 4.**
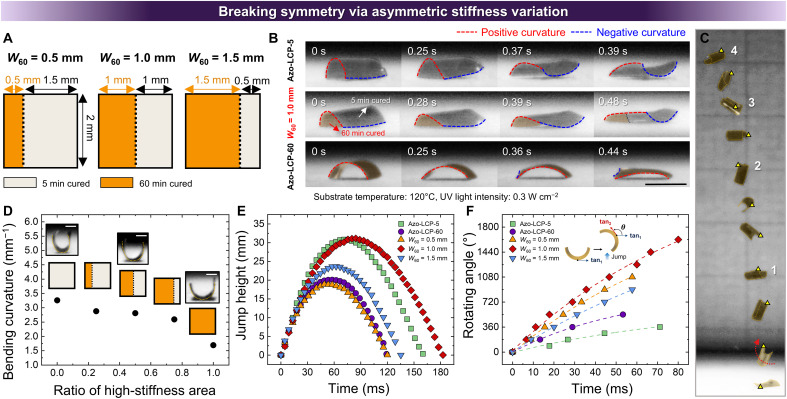
Jumping of Azo-LCPs with asymmetric stiffness variation. (**A**) Schematic representing asymmetrically patterned Azo-LCPs with varied proportions of the rigid region. (**B**) Digital images illustrating topological transition of two uniformly cross-linked Azo-LCPs (Azo-LCP-5 and Azo-LCP-60) and an Azo-LCP film with patterned stiffness variation (*W*_60_ = 1.0 mm). Scale bar, 2 mm. (**C**) Digital images of the sequential rotational jump of asymmetrically patterned Azo-LCP film (*W*_60_ = 1.0 mm). The numbers indicate rotation cycles. Grid, 5 mm. (**D**) Measured UV-induced bending curvatures of Azo-LCPs with uniform cross-linking densities and asymmetrical stiffness variation (corresponding photographs are shown at the top). Scale bars, 0.5 mm. Time-resolved (**E**) jump heights and (**F**) rotating angles of Azo-LCP-5, Azo-LCP-60, and asymmetrically patterned Azo-LCPs with varied ratios of high-stiffness area.

Next, we systematically varied the area ratio between soft and rigid regions of the asymmetric patterns (Fig. 4A). The width of the rigid region was denoted as *W*_60_ and was varied to 0.5, 1.0, and 1.5 mm. A distinctively asymmetrical topological transition was observed in the Azo-LCP with *W*_60_ = 1.0 mm, in contrast to the behavior of uniformly cross-linked Azo-LCPs that showed symmetric anticlastic structure (Fig. 4B and movie S3). During topological transition, the Azo-LCP with *W*_60_ = 1.0 mm induced localized curvatures as sequential deformation initiated in the soft region and propagated toward the rigid region (fig. S17). These different photogenerated strain responsivities cause an asymmetric and biased anticlastic structure. As a result, Azo-LCP with *W*_60_ = 1.0 demonstrated biased and rotational photomechanical jump (Fig. 4C). The strategic combination of soft and rigid regions in a single Azo-LCP body allowed us to develop a model system that harnessed the advantages of Azo-LCP-5 and Azo-LCP-60 while addressing the aforementioned trade-off relationship. As demonstrated in Fig. 4D and fig. S18, the patterned Azo-LCPs exhibited bending curvature values between those of Azo-LCP-5 and Azo-LCP-60, suggesting a higher snap-through energy barrier compared to the fully rigid Azo-LCP-60. Under the condition where *W*_60_ = 1.0 mm, we achieved a maximum jump height of 31 mm, which corresponds to 15.5 times the BL and 1550 times the thickness (Fig. 4E). This jump height is slightly higher than that achieved with the Azo-LCP-5, which had a greater initial curvature. This enhancement is attributed to the rigid region promoting more efficient stress accumulation, while the soft region mitigates excessive reduction in initial curvature. However, *W*_60_ = 0.5 mm and 1.5 mm conditions showed jumping performances similar to those of the fully rigid Azo-LCP-60. At *W*_60_ = 0.5 mm, the rigid region suppressed formation of high initial curvature, while its narrow width resulted in only a minimal increase in stress accumulation. At *W*_60_ = 1.5 mm, the rigid region was too dominant to allow sufficient curvature formation. Both cases failed to overcome the intrinsic trade-off between initial curvature and stiffness.

An asymmetric and sequential topological transition shifted the energy-releasing direction and generated a rotational torque during photomechanical jump. Therefore, as shown in Fig. 4F, the asymmetrically patterned Azo-LCP achieved a higher rotating angle during the jump than the uniformly cross-linked Azo-LCPs. To calculate the rotating angle, we measured the angular difference between tangent vector of the initial negative curvature and that of the curvature observed during the jump. Consequently, Azo-LCP with *W*_60_ = 1.0 mm achieved a maximum rotating angle of 1620°, with an initial angular velocity of 564 mm s^−1^ due to biased energy release (fig. S19). Azo-LCP with *W*_60_ = 1.0 mm achieved a comparable jump height with Azo-LCP-5 despite exhibiting a substantially greater number of rotations during the jump. This result underscores the enhanced stress accumulation achieved by strategic integration of spatially patterned stiffness variation. However, lateral force applied to the Azo-LCP was insufficient to induce a horizontal distance greater than 11.8 mm (fig. S20). Achieving both high vertical jump height and long horizontal jump distance is essential for enhancing overall jumping performance. As lateral force is generated due to tilted energy release, the enhancement of vertical jump height is limited. As the next step, we need to develop distinct stiffness variation patterns that can modulate rotational motion during photomechanical jumping, thereby providing selective amplification of either lateral or vertical force for a long-range directional or high vertical jumping, respectively.

### Directional jump via corner-rigid pattern

**Fig. 5.**
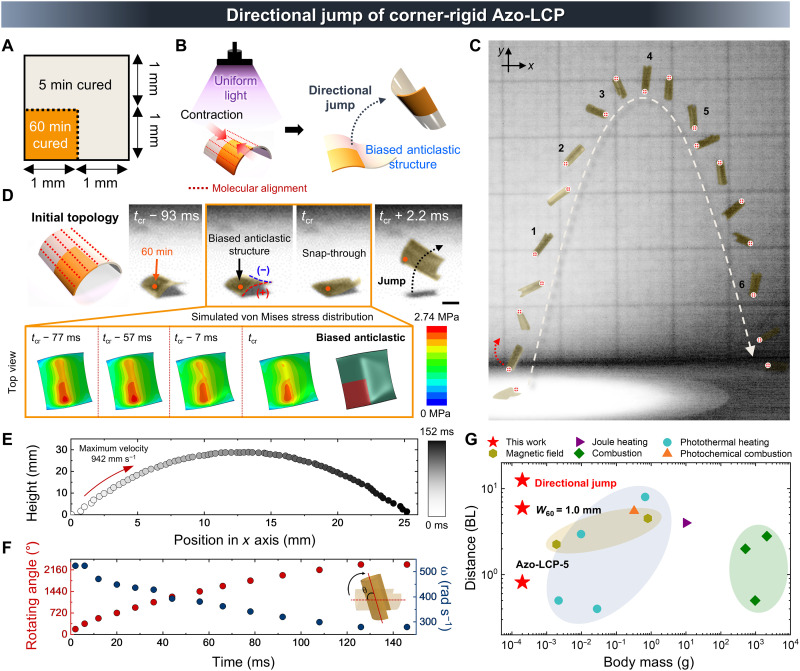
Directional jumping via a corner-rigid pattern. (**A**) Illustration of corner-rigid pattern and (**B**) corresponding topological transition scheme, demonstrating tilted energy release to produce directional jumps. (**C**) Digital images of directional jump of corner-rigid Azo-LCP. Numbers indicate rotation cycles. Grid, 5 mm. (**D**) Digital images illustrating biased take-off induced by an asymmetric topological transition, along with simulation results revealing a nonuniform stress distribution during this process. Scale bar, 1 mm. Color bar indicates von Mises stress. (**E**) Jumping height of corner-rigid Azo-LCP with respect to position on the *x* axis. Color bar indicates time profile during directional jump. (**F**) Time-resolved rotating angle (red dot) and angular velocity (blue dot) of the patterned Azo-LCP. (**G**) Comparison of the jumping distance of corner-rigid Azo-LCP with those of reported untethered jumping soft robots categorized according to actuation type: magnetic field (*29*, *30*), joule heating (*31*), combustion (*32*–*35*), and photothermal heating (*36*–*40*).

As a strategy to increase jumping distance and directional movement, we adopted a corner-rigid pattern that can maximize tilted energy release. Building upon the previous asymmetric pattern, in which heterogeneity was introduced in one dimension, we engineered two-dimensional (2D) heterogeneity by placing the rigid region in one-quarter of the Azo-LCP body, as illustrated in Fig. 5A. In this stiffness variation pattern, the dimension of the rigid area is adequate to establish a monoclastic (+) structure in the corner. In addition, the surrounding areas were loosely cross-linked, allowing a highly biased anticlastic structure to form during topological transition. Thus, a directional jump was induced under uniform light irradiation based on biased topological transition resulting from asymmetric strain responsivity. As illustrated schematically in Fig. 5B, the soft region initially deforms along molecular alignment, whereas the rigid region deforms more slowly. Once reaching the energy barrier for snap-through, a topological transition occurs in the high-stiffness area, and the asymmetric energy release triggers a tilted directional jump as demonstrated in Fig. 5C and movie S4. Because of the enhanced rotational force caused by a further shift in energy release away from the center, the jump was accompanied by a rapid rotational motion, and the sample completed a maximum of six 360° rotations along the trajectory. The experimentally observed topological transition confirmed a biased anticlastic structure leading to a directional jump from rigid to soft region; nonuniform deformation due to heterogeneity in stiffness is supported by simulation results produced using a photomechanical continuum model (Fig. 5D and movie S5) (*25*, *26*). As a result, the corner-rigid Azo-LCP demonstrated a jump distance of 25 mm (12.5 times the BL) with a jump height of 28.7 mm (14.4 times the BL). Notably, the jump height of corner-rigid Azo-LCP is comparable to that of the fully soft Azo-LCP-5 (Fig. 5E). Furthermore, it achieved a maximum rotating angle of 2340° and an angular velocity of 523 mm s^−1^ (Fig. 5F). The jump distance of corner-rigid Azo-LCP demonstrates clear superiority over other reported lightweight untethered soft jumping robot systems (Fig. 5G and table S3). This example highlights the potential of patterned stiffness variation in overcoming fundamental trade-offs and achieving efficient snap-through–driven actuation.

### Enhanced vertical jumping via center-rigid pattern

Although asymmetric stiffness variation is an effective strategy for imparting directionality to motion, the biased anticlastic structure results in stress dissipation during the energy-accumulation process. To increase stress-accumulation efficiency, symmetric patterns should be used, following which the anticlastic structure exhibits an in-plane circular symmetry. Considering this aspect of symmetry, we implemented a center-rigid pattern to enhance stress accumulation and increase vertical component of force.

**Fig. 6.**
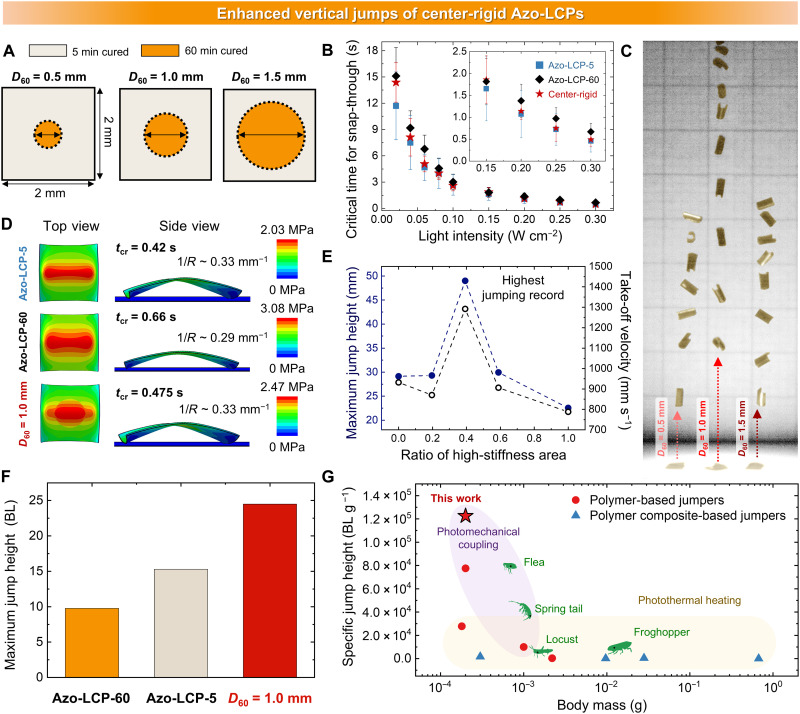
Enhanced vertical jumping via a center-rigid pattern. (**A**) Schematic representation of the adopted spatial pattern of stiffness variation and (**B**) *t*_cr_ of Azo-LCP-5, Azo-LCP-60, and center-rigid Azo-LCP (*D*_60_ = 1 mm) as a function of UV light intensities. (**C**) Digital images of upward jumping of center-rigid Azo-LCPs. Grid, 5 mm. (**D**) Simulated von Mises stress distribution and anticlastic structures of Azo-LCP-5, Azo-LCP-60, and center-rigid Azo-LCP with *D*_60_ = 1.0 mm. (**E**) Comparison of jumping heights and take-off velocities of Azo-LCPs with different ratios of high-stiffness area. Comparison of the jumping heights of Azo-LCPs with (**F**) different mechanical stiffness modulations, (**G**) living species (*41*–*44*), and reported light-driven untethered polymeric jumping robots categorized by material types [single materials (*9*, *38*, *45*, *46*) and polymer composites (*36*, *37*, *39*, *40*, *47*)].

**Fig. 7.**
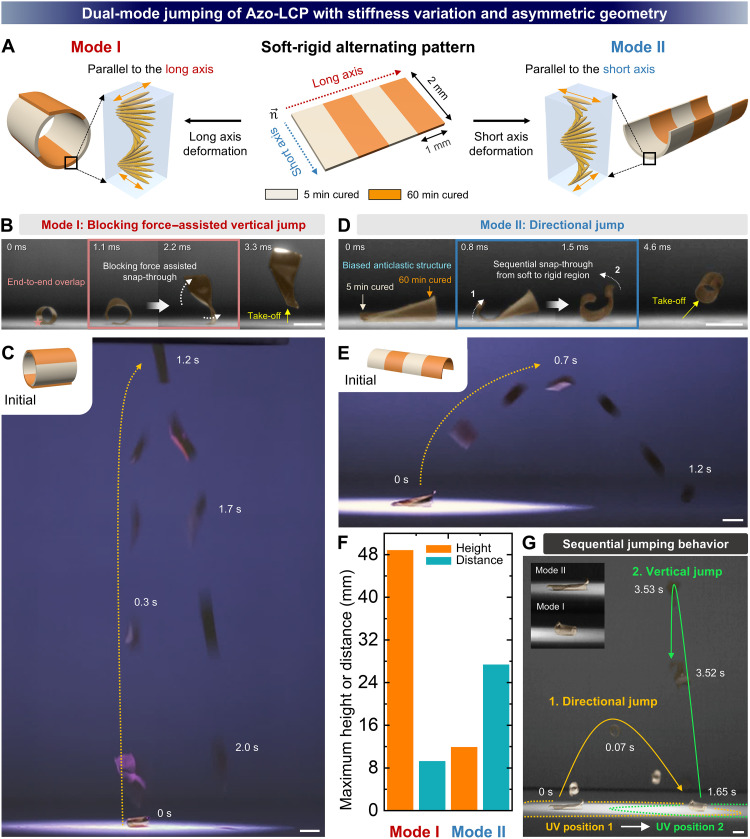
Dual jumping modes of Azo-LCPs with a soft-rigid alternating pattern and high aspect ratio asymmetric geometry. (**A**) Schematic illustration showing distinct photoinduced deformation modes of the dual-mode Azo-LCP jumper with an aspect ratio of 2. Bending along the long axis corresponds to Mode I, while bending along the short axis corresponds to Mode II. (**B**) Photomechanical energy storage and release mechanism of Mode I, in which the blocked force increases energy barrier during snap-through. (**C**) Digital image showing vertical jumping based on Mode I deformation. (**D**) Asymmetric snap-through behavior of Mode II, induced by alternating soft-rigid pattern. (**E**) Digital image showing directional jumping behavior based on Mode II deformation. (**F**) Comparison of maximum jump heights and distances achieved by Mode I and II jumping. (**G**) Sequential jumping behavior, with a Mode II jump followed by a Mode I jump. Scale bars, 2 mm.

As shown in Fig. 6A, a 60 min–cured rigid region was patterned as a circular area at the center, surrounded by a 5 min–cured soft region. The center-rigid area diameter is denoted as *D*_60_. When comparing the center-rigid Azo-LCP (*D*_60_ = 1.0 mm) with uniformly cross-linked Azo-LCPs, we initially expected its photomechanical strain rate to fall between the two uniformly cross-linked Azo-LCPs due to the inclusion of a central rigid region. The *t*_cr_ and threshold UV intensity, required to initiate jumps in the center-rigid Azo-LCP, exhibit intermediate values between those of Azo-LCP-5 and Azo-LCP-60 (Fig. 6B and figs. S21 and S22). However, center-rigid Azo-LCP had an average *t*_cr_ of 0.49 s under 0.3 W cm^−2^ UV exposure, which is only 0.03 s longer than that of Azo-LCP-5 (0.46 s) and 0.18 s shorter than Azo-LCP-60 (0.67 s). This corresponds to only a 6% increase compared to Azo-LCP-5, but a substantial 37% reduction compared to Azo-LCP-60, despite the center-rigid Azo-LCP containing a rigid region that accounts for ~40% of the total volume. These results indicate that the incorporation of the rigid region has only a minimal effect on reducing photomechanical strain rate. Notably, the center-rigid Azo-LCP demonstrated a substantial increase in the bending curvature to 4.5 mm^−1^, which is 40.6% greater than that of Azo-LCP-5 (fig. S23). In addition, we investigated the effect of *D*_60_ by varying it to 0.5, 1.0, and 1.5 mm. However, both *D*_60_ = 0.5 and 1.5 mm did not show an improvement in the UV-induced bending curvature and even exhibited lower values compared to Azo-LCP-5. In a uniformly cross-linked sample, a global curvature develops during topological transition, accompanied by a uniform strain response across the entire structure. Therefore, in this case, the curvature is determined solely by the mechanical stiffness of the material. In contrast, the introduction of stiffness variation induces greater localized curvature as a result of the mechanical discontinuity at the interface between soft and rigid regions. This promotes effective stress accumulation as well as generation and preservation of high initial curvature of monoclastic (+) structure, supported by a rigid center. However, if the center-rigid area is too small, it fails to generate a sufficient localized curvature due to the smaller interface between soft and rigid regions, whereas an overly large rigid region hinders formation of a higher initial curvature. As a result, the configuration with *D*_60_ = 1.0 mm yields the largest initial curvature and, consequently, achieves the greatest jump height among all tested patterns (Fig. 6C). The simulated von Mises stress distribution in Fig. 6D confirms that center-rigid Azo-LCP has larger stress (2.47 MPa) than uniformly cross-linked Azo-LCP-5 (2.03 MPa), with stress concentration near the interfaces. In addition, the simulated anticlastic structure of center-rigid Azo-LCP has a higher curvature than Azo-LCP-60. These findings provide direct evidence that this center-rigid patterning strategy is effective in achieving enhanced vertical jump performance. A high initial curvature effectively enhances the snap-through energy barrier, while a fast photomechanical strain rate minimizes energy loss during transition from monoclastic (+) to anticlastic geometry, thereby enabling efficient photomechanical jumping. As evident in Fig. 7E and movie S6, Azo-LCP with *D*_60_ = 1.0 mm demonstrated remarkable jumping performance compared to uniformly cross-linked Azo-LCPs and other center-rigid Azo-LCPs with different *D*_60_. Furthermore, the inversely patterned design exhibited a jumping performance comparable to that of Azo-LCP-60, as the high-stiffness region dominated the overall curvature formation, further supporting our previous claims (fig. S24). Notably, Azo-LCP with *D*_60_ = 1.0 mm recorded a maximum jump height of 49 mm, which corresponds to 24.5 times its BL and 2450 times its thickness, and a maximum instantaneous velocity of ~1170 mm s^−1^. This jump height is about 1.6 and 2.5 times higher than that of Azo-LCP-5 and Azo-LCP-60, respectively (Fig. 6F and fig. S25). To evaluate energy conversion efficiency (η) of the photomechanical jump, we calculated efficiency as the percent ratio of input UV light energy to the maximum gravitational potential energy generated during the jump (Eq. 2)

Energy conversion efficiency(η,%)=Epotential energyEinput light energy=m×g×hI×A×t×100 (2)

where *I* is UV light intensity, *A* is irradiated area of Azo-LCP, *t* is exposure time until snap-through, which corresponds to *t*_cr_, *m* is mass of Azo-LCP, *g* is gravitational acceleration, and *h* is the maximum jump height. As light intensity increased, the center-rigid Azo-LCP exhibited a decrease in *t*_cr_ and a corresponding increase in jump height, resulting in an overall rise in energy conversion efficiency (fig. S26A). Above a light intensity of 0.25 W cm^−2^, the rate of decrease in *t*_cr_ with increasing light intensity becomes markedly smaller, leading maximum energy conversion efficiency to reach saturation. Maximum energy conversion efficiency of the center-rigid Azo-LCP is 1.4 and 3.1 times higher than that of Azo-LCP-5 (8.9%) and Azo-LCP-60 (4.0%), respectively (fig. S26B). This result highlights the finding that hybrid structure, composed of both rigid and soft regions, surpasses the performance limits of uniformly cross-linked systems having intrinsic trade-off relationships. As a result, our stiffness-patterning strategy enabled the LCP jumper to achieve a superior specific jump height compared with those of other light-driven untethered polymeric jumping robots and centimeter-scale living organisms (Fig. 6G and tables S3 and S4).

### Dual-mode jumping via patterned stiffness variation and asymmetric geometry

Previously, we demonstrated that placing a rigid region at the corner and center of Azo-LCP enabled direction control and enhanced jump height, respectively, during photomechanical jumping. However, there exists an intrinsic trade-off between jump height and distance. When snap-through occurs at a tilted angle, energy release is also tilted, which reduces the vertical force component. This results in a lower jump height but longer horizontal distance. Hence, center-rigid patterns are advantageous for achieving high vertical jumps, whereas corner-rigid patterns are more suitable for enhancing jump distance. This presents a limitation where each pattern yields only one type of advantageous behavior. To address this limitation, we integrated a geometrically asymmetric design with stiffness variation to enable dual-mode actuation within a single Azo-LCP. While a square design (2 mm by 2 mm; aspect ratio = 1) has been used up to this point, we have extended it to a rectangular design with an aspect ratio greater than 1 (Fig. 7A). Furthermore, we introduced a soft-rigid alternating pattern along the long axis to intentionally mismatch UV reactivity between the two ends of the strip. This design enables Azo-LCP to exhibit two distinct deformation modes: Mode I, based on long-axis bending, and Mode II, based on short-axis bending, due to the 270° STN alignment providing an orthogonal alignment between top and bottom surfaces.

The dual-mode jump was first demonstrated using Azo-LCP with soft-rigid alternating pattern and an aspect ratio of 2. In Mode I deformation, the LC alignment is parallel to the long axis, and Azo-LCP forms a rolled-up structure with both ends overlapping when UV light is irradiated onto its surface (Fig. 7B). This end-to-end overlap generates an additional blocking force, which enhances snap-through energy barrier. This behavior is facilitated by an extended BL compared to the previous design with an aspect ratio of 1. During formation of the rolled-up structure, the 5 min–cured region bends earlier than the 60 min–cured region due to its faster photomechanical strain response (fig. S27). Immediately after, the center of mass of the partially bent Azo-LCP shifts toward the 60 min–cured region causing the film to tip over. As the inner side of the 60 min–cured region becomes exposed to light, it begins to bend more slowly, eventually forming a rolled-up structure where the rigid region folds over the soft region. In addition, when Azo-LCP tips over, the bottom surface with positive Gaussian curvature is exposed to UV light, gradually deforming into a localized anticlastic structure and accumulating elastic energy. As a result, Azo-LCP with an aspect ratio of 2 produced a remarkable vertical jump height of 48.8 mm via Mode I deformation (Fig. 7C and movie S7). Meanwhile, Mode II deformation induced directional photomechanical jumping by sequential snap-through from soft to rigid region, driven by the formation of a biased anticlastic structure (Fig. 7D). Unlike Mode I, the shorter width of the bent ends prevents end-to-end overlap, resulting in a snap-through mechanism similar to that observed in the previous aspect ratio 1 design. First, topological transition initiates from a monoclastic (+) structure elongated along the long axis. The stiffness mismatch across the alternating pattern induces a longitudinal strain gradient, triggering an earlier topological transition into an anticlastic structure at the softer end. As a result, a snap-through propagates sequentially from soft to rigid region with a temporal delay, which generates an asymmetric and directional energy release. This biased energy release ultimately drives directional jumping from soft to rigid region, with a probability of 80.6% based on more than 30 independent trials (fig. S28). Moreover, Mode II deformation also resulted in a maximum horizontal distance of 27.3 mm (Fig. 7E and movie S8). These performances are comparable to those of the vertical jump height of center-rigid Azo-LCP (49 mm) and horizontal jump distance of corner-rigid Azo-LCP (25 mm), despite being achieved by a single body with nearly twice the body mass (Fig. 7F). A summary of jumping performance across uniformly cross-linked, single-mode, and dual-mode jumpers is highlighted in Table 1.

**Table 1.** Jumping performance of Azo-LCP jumpers.

Azo-LCP type	Mass (mg)	Maximum height (mm)	Maximum distance (mm)	Direction control	Jump mode tunability
Uniformly cross-linked	0.2	29.1	1.6	Χ	Χ
Single-mode (corner-rigid)	0.2	28.7	25.0	○	Χ
Single-mode (center-rigid)	0.2	49.0	7.3	Χ	Χ
Dual-mode	0.4	48.8	27.3	○	○

We highlight that dual-mode jumping behavior results from the synergistic effect of integrating both asymmetric geometry and soft-rigid alternating pattern. As shown in fig. S29 (A and B), Mode I and Mode II deformations were examined in Azo-LCP-5 and Azo-LCP-60 samples with an aspect ratio of 2. In Mode I, although Azo-LCP-5 exhibited an end-to-end overlap and formed a rolled-up structure, it failed to overcome a newly added blocking force due to the intrinsically limited stress accumulation capacity of its soft body. In the case of Azo-LCP-60, its excessively high stiffness prevents formation of the end-to-end overlap. Hence, blocking force–assisted vertical jump was not achieved in either case. In Mode II deformation, both uniformly cross-linked Azo-LCPs exhibited photomechanical jumping. However, because snap-through occurred symmetrically, directional jumping was not achieved. We also systematically investigated the effect of aspect ratio on the jumping behavior of Azo-LCPs with soft-rigid alternating patterns. At an aspect ratio of 3, both Mode I and Mode II deformations were observed, corresponding to vertical and directional jumps, respectively (fig. S30A). However, at an aspect ratio of 4, only directional jumps derived from Mode II deformation were observed (fig. S30B). This is most likely because at high aspect ratios, the edge-to-edge overlap formed in Mode I becomes excessively long, resulting in a blocking force too large to overcome with the available photogenerated strain. In comparing jumping performance between aspect ratios 2 and 3, we found that blocking force–assisted jump height markedly decreased at an aspect ratio of 3 (fig. S31A). In addition, the difference between jump height and distance was substantially narrowed. This trend can be attributed to the increase in mass and the further tilting of energy release direction caused by enhanced geometric asymmetry at higher aspect ratios. Directional jumping performance also showed a notable decline, which we attribute primarily to the increased mass (fig. S31B). Last, Fig. 7G, fig. S32, and movie S9 demonstrate sequential and consecutive jumping of the dual-mode Azo-LCP jumper, in which Mode I and Mode II jumps occurred consecutively under continuous UV irradiation. This design strategy highlights the potential of patterning stiffness variation to tune the snap-through energy barrier, allowing control over both jumping performance and direction, while engineered geometric asymmetry further enhances functionality.

## DISCUSSION

We used a facile yet robust strategy of patterning stiffness variation in photoresponsive polymer films to program its local stress-strain responsivity and achieve versatile jumps without modifying the physical form factor, chemical composition, or the external stimulus. Through spatially encoded stiffness profiles, asymmetrical corner-rigid and symmetrical center-rigid patterns were introduced into Azo-LCP monoliths to induce directional jumping via biased curvature and to maximize vertical jump height through enhanced elastic energy storage, respectively. Asymmetrical stiffness variation led to temporally resolved sequential deformation, which generated rotational torques and tilted energy release. These effects enabled directional jumping under uniform light irradiation, achieving a maximum rotating angle of 2340° and an initial angular velocity of 564 mm s^−1^. The corner-rigid Azo-LCP exhibited a maximum horizontal jump distance of 25 mm (12.5 times its BL). In contrast, the center-rigid Azo-LCP effectively maintained initial curvature and promoted efficient stress accumulation, reaching a vertical jump height of 49 mm (24.5 times BL). This record is 145% greater than that of the uniformly cross-linked system and surpasses the performance of biological jumpers such as fleas and spring tails. Last, the integration of geometric asymmetry with a soft-rigid alternating pattern enabled a single Azo-LCP to sequentially perform both directional and vertical dual-mode jumps. The dual-mode Azo-LCP jumper with an aspect ratio of 2 achieved a vertical jump height of 48.8 mm and a horizontal jump distance of 27.3 mm. These values are comparable to those of single-mode corner-rigid and center-rigid Azo-LCPs with an aspect ratio of 1, despite the dual-mode jumper having nearly twice the mass. The strategy of combining patterned stiffness variation and geometric asymmetry demonstrated here provides a scalable and broadly applicable design principle to enhance nonlinear dynamics and multifunctionality of soft actuators under diverse stimuli.

## MATERIALS AND METHODS

### Materials

The photoresponsive molecular switch, 4,4′-bis[6-(acryoloxy)hexyloxy] azobenzene (2-azo), and LC monomer, 4-methoxybenzoic acid 4-(6-acryloyloxy-hexyloxy)phenyl ester (RM105), were purchased from SYNTHON Chemicals. The LC comonomer, 4-(3-acryloyloxypropyloxy)-benzoic acid 2-methyl-1,4-phenylene ester (RM257), and the chiral dopant, *R*-octan-2-yl 4-{[4-(hexyloxy)benzoyl]oxy}benzoate (R811), were procured from Merck. IRGACURE 784 (I-784), used as a photoinitiator, was purchased from Ciba. All commercial reagents were used as received, and their chemical structures are shown in fig. S3.

### Preparation of Azo-LCP films

An Azo-LCP mixture was prepared by mechanically mixing 10 wt % of RM105, 68 wt % of RM257, 20 wt % of 2-azo, 0.25 wt % of R811, and 1.75 wt % of I-784. The mixed materials were subsequently heated to 110°C and injected into a glass cell using capillary force. Different LC monomers were mixed to lower the melting temperature of the mixture and reduce the risk of premature thermal polymerization (*27*, *28*). Glass slides with a polyamide (Elvamide, DuPont) coating were mechanically rubbed with microfiber cloth to prepare an alignment layer. Two glass slides with orthogonal alignment were then assembled with a gap of 20 μm. The amount of the chiral dopant required to induce 270° STN alignment was calculated according to the thickness of the LCP film. To polymerize the Azo-LCP molten mixture in nematic phase, cell temperature was decreased from 95° to 69°C and maintained at 69°C during the photopolymerization process; this temperature corresponds to that between the melting temperature (*T*_m_) and nematic-to-isotropic phase transition temperature (*T*_NI_). The Azo-LCP mixture was then photopolymerized using 532-nm light (intensity: 50 mW cm^−2^) from a light-emitting diode. After being photocured, the Azo-LCP films were harvested from the glass cells.

### Characterization of thermomechanical properties and stress-strain behaviors

The thermomechanical properties of the Azo-LCP films were determined by a temperature ramp test using stress-controlled DMA (DMA Q800, TA Instruments). The temperature ramp test was conducted with a heating rate of 3°C min^−1^ and 1-Hz frequency dynamic and heating rate. The glass-transition temperature, *T*_g,_ was determined as the temperature corresponding to the maximum peak of tan δ. The cross-linking density was calculated using Flory’s rubber elasticity theory (*22*). Stress-strain curves of Azo-LCPs with different cross-linking densities were measured using a strain-controlled DMA (RSA-G2, TA Instruments). Uniaxial tensile tests were performed at a strain rate of 0.001 mm s^−1^.

### Characterization of snap-through topological transition and photomechanical jumping

To prevent Gaussian intensity profile and ensure uniform light irradiation, the UV source was equipped with a collimator. Light intensity was varied from 0.02 to 0.3 W cm^−2^ and was measured by an optical power meter (PM100D, Thorlabs). The light intensity profile is shown in fig. S33. For jumping actuation, Azo-LCP films were placed on a hot plate, and their temperatures were varied from 70° to 130°C. A thermal imaging camera (E40, FLIR) was used to monitor temperature variations in the Azo-LCP film while it was heated, either on a hot plate or through photothermal effects via photoirradiation. The photomechanical jumping of the Azo-LCP film under narrowband UV-light (365 nm) irradiation (S2000, OmniCure) was captured using a mirrorless digital camera and a high-speed camera (C110, Phantom). Finite element simulations were conducted using the Expanse compute cluster (award TG-MSS170004) in the Advanced Cyberinfrastructure Coordination Ecosystem: Services and Support (ACCESS). Simulation details are provided in the Supplementary Materials (fig. S34 and table S5).

## Supplementary Materials

### The PDF file includes:

Supplementary Text Figs. S1 to S34 Tables S1 to S5 Legend for movies S1 to S9 References

## References

[1] M. L. Smith, G. M. Yanega, A. Ruina, Elastic instability model of rapid beak closure in hummingbirds. J. Theor. Biol. 282, 41–51 (2011). 21609721 10.1016/j.jtbi.2011.05.007

[2] Y. Wang, Q. Wang, M. Liu, Y. Qin, L. Cheng, O. Bolmin, M. Alleyne, A. Wissa, R. H. Baughman, D. Vella, S. Tawfick, Insect-scale jumping robots enabled by a dynamic buckling cascade. Proc. Natl. Acad. Sci. U.S.A. 120, e2210651120 (2023). 36689664 10.1073/pnas.2210651120 PMC9945960

[3] Y. Forterre, J. M. Skotheim, J. Dumals, L. Mahadevan, How the Venus flytrap snaps. Nature 433, 421–425 (2005). 15674293 10.1038/nature03185

[4] Y. Kim, J. van den Berg, A. J. Crosby, Autonomous snapping and jumping polymer gels. Nat. Mater. 20, 1695–1701 (2021). 33526877 10.1038/s41563-020-00909-w

[5] Y. Luo, D. K. Patel, Z. Li, Y. Hu, H. Luo, L. Yao, C. Majidi, Intrinsically multistable soft actuator driven by mixed-mode snap-through instabilities. Adv. Sci. 11, e2307391 (2024). 10.1002/advs.202307391 PMC11095224 38447200

[6] B. Gorissen, D. Melancon, N. Vasios, M. Torbati, K. Bertoldi, Inflatable soft jumper inspired by shell snapping. Sci. Robot. 5, eabb1967 (2020). 33022625 10.1126/scirobotics.abb1967

[7] T. S. Hebner, K. Korner, C. N. Bowman, K. Bhattacharya, T. J. White, Leaping liquid crystal elastomers. Sci. Adv. 9, eade1320 (2023). 36652507 10.1126/sciadv.ade1320 PMC9848472

[8] Y. Yang, Y. Wang, Snapping for 4D-printed insect-scale metal-jumper. Adv. Sci. 11, 2307088 (2024). 10.1002/advs.202307088 PMC10797476 37997200

[9] J. Jeon, J. C. Choi, H. Lee, W. Cho, K. Lee, J. G. Kim, J. W. Lee, K. Il Joo, M. Cho, H. R. Kim, J. J. Wie, Continuous and programmable photomechanical jumping of polymer monoliths. Mater. Today 49, 97–106 (2021).

[10] A. D. Shaw, S. A. Neild, D. J. Wagg, P. M. Weaver, A. Carrella, A nonlinear spring mechanism incorporating a bistable composite plate for vibration isolation. J. Sound Vib. 332, 6265–6275 (2013).

[11] M. Gomez, D. E. Moulton, D. Vella, Critical slowing down in purely elastic ‘snap-through’ instabilities. Nat. Phys. 13, 142–145 (2017).

[12] J. J. Wie, M. R. Shankar, T. J. White, Photomotility of polymers. Nat. Commun. 7, 13260 (2016). 27830707 10.1038/ncomms13260 PMC5109552

[13] W. Cho, J. Jeon, W. Eom, J. G. Lee, D. G. Kim, Y. S. Kim, T. H. Han, J. J. Wie, Photo-triggered shape reconfiguration in stretchable reduced graphene oxide-patterned azobenzene-functionalized liquid crystalline polymer networks. Adv. Funct. Mater. 31, 2102106 (2021).

[14] W. Feng, Q. He, L. Zhang, Embedded physical intelligence in liquid crystalline polymer actuators and robots. Adv. Mater. 37, 2312313 (2024). 38375751 10.1002/adma.202312313 PMC11733722

[15] J. G. Kim, J. Jeon, R. Sivakumar, J. Lee, Y. H. Kim, M. Cho, J. H. Youk, J. J. Wie, Light-fueled climbing of monolithic torsional soft robots via molecular engineering. Adv. Intell. Syst. 4, 2100148 (2022).

[16] J.-C. Choi, J. Jeon, J.-W. Lee, A. Nauman, J. G. Lee, W. Cho, C. Lee, Y.-M. Cho, J. J. Wie, H.-R. Kim, Steerable and agile light-fueled rolling locomotors by curvature-engineered torsional torque. Adv. Sci. 10, 2304715 (2023). 10.1002/advs.202304715 PMC10602523 37565602

[17] C. Ma, Y. Zhang, S. Jiao, M. Liu, Snap-through of graphene nanowrinkles under out-of-plane compression. Nanotechnology 34, 015705 (2023). 10.1088/1361-6528/ac9418 36137514

[18] R. M. Springman, J. L. Bassani, Snap transitions in adhesion. J. Mech. Phys. Solids 56, 2358–2380 (2008).

[19] M. Ravi Shankar, M. L. Smith, V. P. Tondiglia, K. M. Lee, M. E. McConney, D. H. Wang, L. S. Tan, T. J. White, Contactless, photoinitiated snap-through in azobenzene-functionalized polymers. Proc. Natl. Acad. Sci. U.S.A. 110, 18792–18797 (2013). 24190994 10.1073/pnas.1313195110 PMC3839691

[20] K. Yang, S. Won, J. E. Park, J. Jeon, J. J. Wie, Magnetic swarm intelligence of mass-produced, programmable microrobot assemblies for versatile task execution. Device 3, 100626 (2025).

[21] H. Moon, J. G. Lee, W. Cho, J. Jeon, J. E. Park, J. J. Wie, Structure-property-actuation relationships of shape-fixable magnetic vitrimer micropillar arrays. Sens. Actuators B Chem. 402, 135092 (2024).

[22] J. Jeon, H. Moon, J. Park, S. Won, J. E. Park, Z. Ku, J. O. Kim, J. J. Wie, Collective and rapid high amplitude magnetic oscillation of anisotropic micropillar arrays. ACS Nano 19, 9946–9957 (2025). 40050612 10.1021/acsnano.4c15987

[23] P. J. Flory, Molecular theory of rubber elasticity. Polym. J. 20, 1–12 (1985).

[24] H. M. D. Bandara, S. C. Burdette, Photoisomerization in different classes of azobenzene. Chem. Soc. Rev. 41, 1809–1825 (2012). 22008710 10.1039/c1cs15179g

[25] K. Korner, A. S. Kuenstler, R. C. Hayward, B. Audoly, K. Bhattacharya, A nonlinear beam model of photomotile structures. Proc. Natl. Acad. Sci. U.S.A. 117, 9762–9770 (2020). 32300009 10.1073/pnas.1915374117 PMC7211941

[26] F. Dadgar-Rad, M. M. Mahjoub, M. Hossain, A hyperelastic beam model for the photo-induced response of nematic liquid crystal elastomers. Extrem. Mech. Lett. 72, 102233 (2024).

[27] D. Liu, D. J. Broer, Liquid crystal polymer networks: Preparation, properties, and applications of films with patterned molecular alignment. Langmuir 30, 13499–13509 (2014). 24707811 10.1021/la500454d

[28] D. J. Broer, R. G. Gossink, R. A. M. Hikmet, Oriented polymer networks obtained by photopolymerization of liquid-crystalline monomers. Angew. Makromol. Chem. 183, 45–66 (1990).

[29] J. Wang, D. Wang, A lightweight jumping robot with untethered actuation. Lect. Notes Comput. Sci. 14270, 71–82 (2023).

[30] W. Hu, G. Z. Lum, M. Mastrangeli, M. Sitti, Small-scale soft-bodied robot with multimodal locomotion. Nature 554, 81–85 (2018). 29364873 10.1038/nature25443

[31] Z. Zhakypov, K. Mori, K. Hosoda, J. Paik, Designing minimal and scalable insect-inspired multi-locomotion millirobots. Nature 571, 381–386 (2019). 31292552 10.1038/s41586-019-1388-8

[32] C. A. Aubin, R. H. Heisser, O. Peretz, J. Timko, J. Lo, E. F. Helbling, S. Sobhani, A. D. Gat, R. F. Shepherd, Powerful, soft combustion actuators for insect-scale robots. Science 381, 1212–1217 (2023). 37708265 10.1126/science.adg5067

[33] M. T. Tolley, R. F. Shepherd, M. Karpelson, N. W. Bartlett, K. C. Galloway, M. Wehner, R. Nunes, G. M. Whitesides, R. J. Wood, “An untethered jumping soft robot” in *2014 IEEE/RSJ International Conference on Intelligent Robots and Systems* (IEEE, 2014), pp. 561–566.

[34] N. W. Bartlett, M. T. Tolley, J. T. B. Overvelde, J. C. Weaver, B. Mosadegh, K. Bertoldi, G. M. Whitesides, R. J. Wood, A 3D-printed, functionally graded soft robot powered by combustion. Science 349, 161–165 (2015). 26160940 10.1126/science.aab0129

[35] M. Loepfe, C. M. Schumacher, U. B. Lustenberger, W. J. Stark, An untethered, jumping roly-poly soft robot driven by combustion. Soft Robot. 2, 33–41 (2015).

[36] C. Ahn, X. Liang, S. Cai, Bioinspired design of light-powered crawling, squeezing, and jumping untethered soft robot. Adv. Mater. Technol. 4, 1900185 (2019).

[37] W. Cho, D. J. Kang, M. J. Hahm, J. Jeon, D. G. Kim, Y. S. Kim, T. H. Han, J. J. Wie, Multi-functional locomotion of collectively assembled shape-reconfigurable electronics. Nano Energy 118, 108953 (2023).

[38] H. Guo, A. Priimagi, H. Zeng, Optically controlled latching and launching in soft actuators. Adv. Funct. Mater. 32, 2108919 (2022).

[39] J. Hu, Z. Nie, M. Wang, Z. Liu, S. Huang, H. Yang, Springtail-inspired light-driven soft jumping robots based on liquid crystal elastomers with monolithic three-leaf panel fold structure. Angew. Chem. Int. Ed. 62, e202218227 (2023). 10.1002/anie.202218227 36624053

[40] Y. Hu, J. Liu, L. Chang, L. Yang, A. Xu, K. Qi, P. Lu, G. Wu, W. Chen, Y. Wu, Electrically and sunlight-driven actuator with versatile biomimetic motions based on rolled carbon nanotube bilayer composite. Adv. Funct. Mater. 27, 1704388 (2017).

[41] G. P. Sutton, M. Burrows, Biomechanics of jumping in the flea. J. Exp. Biol. 214, 836–847 (2011). 21307071 10.1242/jeb.052399

[42] S. Sudo, T. Kainuma, T. Yano, A. Shirai, T. Hayase, Jumps of water springtail and morphology of the jumping organ. J. Jpn. Soc. Exp. Mech. 15, s117–s124 (2015).

[43] X. Mo, D. Romano, M. Miraglia, W. Ge, C. Stefanini, Effect of substrates’ compliance on the jumping mechanism of locusta migratoria. Front. Bioeng. Biotechnol. 8, 661 (2020). 32775320 10.3389/fbioe.2020.00661 PMC7381386

[44] M. Burrows, Jumping performance of froghopper insects. J. Exp. Biol. 209, 4607–4621 (2006). 17114396 10.1242/jeb.02539

[45] H. Arazoe, D. Miyajima, K. Akaike, F. Araoka, E. Sato, T. Hikima, M. Kawamoto, T. Aida, An autonomous actuator driven by fluctuations in ambient humidity. Nat. Mater. 15, 1084–1089 (2016). 27429210 10.1038/nmat4693

[46] B. Lei, Z. Y. Wen, H. K. Wang, J. Gao, L. J. Chen, Bioinspired jumping soft actuators of the liquid crystal elastomer enabled by photo-mechanical coupling. ACS Appl. Mater. Interfaces 16, 1596–1604 (2024). 38153381 10.1021/acsami.3c16530

[47] H. Li, J. Wang, Ultrafast yet controllable dual-responsive all-carbon actuators for implementing unusual mechanical movements. ACS Appl. Mater. Interfaces 11, 10218–10225 (2019). 30793583 10.1021/acsami.8b22099

[48] D. Liu, D. J. Broer, New insights into photoactivated volume generation boost surface morphing in liquid crystal coatings. Nat. Commun. 6, 8334 (2015). 26388022 10.1038/ncomms9334 PMC4595720

[49] T. J. White, D. J. Broer, Programmable and adaptive mechanics with liquid crystal polymer networks and elastomers. Nat. Mater. 14, 1087–1098 (2015). 26490216 10.1038/nmat4433

[50] U. Hrozhyk, S. Serak, N. Tabiryan, T. J. White, T. J. Bunning, Bidirectional photoresponse of surface pretreated azobenzene liquid crystal polymer networks. Opt. Express 17, 716–722 (2009). 19158885 10.1364/oe.17.000716

