# Supplementary material for: Spatially patterned stiffness variation in a light-triggered jumper for symmetry breaking and high snap-through efficiency
Source: Sci Adv. 2025 Aug 29;11(35):eadx8301. doi: 10.1126/sciadv.adx8301 (PMC12396311; doi:10.1126/sciadv.adx8301)
Supplement: Supplementary file 1 — Supplementary Text Figs. S1 to S34 Tables S1 to S5 Legend for movies S1 to S9 References [file sciadv.adx8301_sm.pdf]

Science Advances
AAAS

Supplementary Materials for

Spatially patterned stiffness variation in a light-triggered jumper for symmetry breaking and high snap-through efficiency

Min Jeong Hahm *et al.*

Corresponding author: Jeong Jae Wie, jjwie@hanyang.ac.kr

Sci. Adv. **11**, eadx8301 (2025)
DOI: 10.1126/sciadv.adx8301

The PDF file includes:

Figs. S1 to S34
Supplementary Text
Tables S1 to S5
Legend for movies S1 to S9
References

Other Supplementary Material for this manuscript includes the following:

Movies S1 to S9

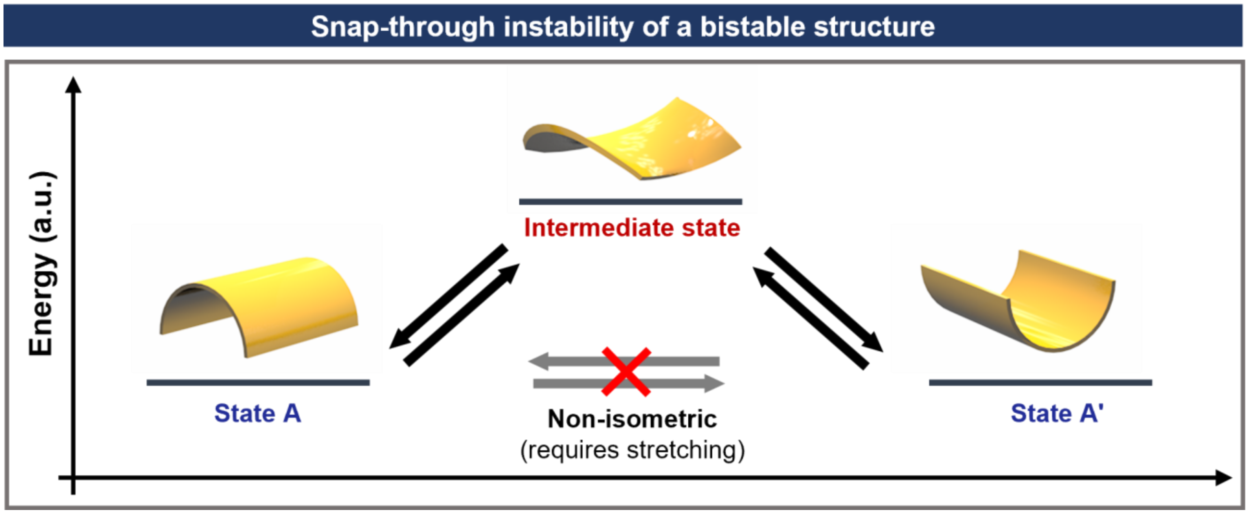

Fig. S1. The potential energy diagram for non-isometric and isometric states.

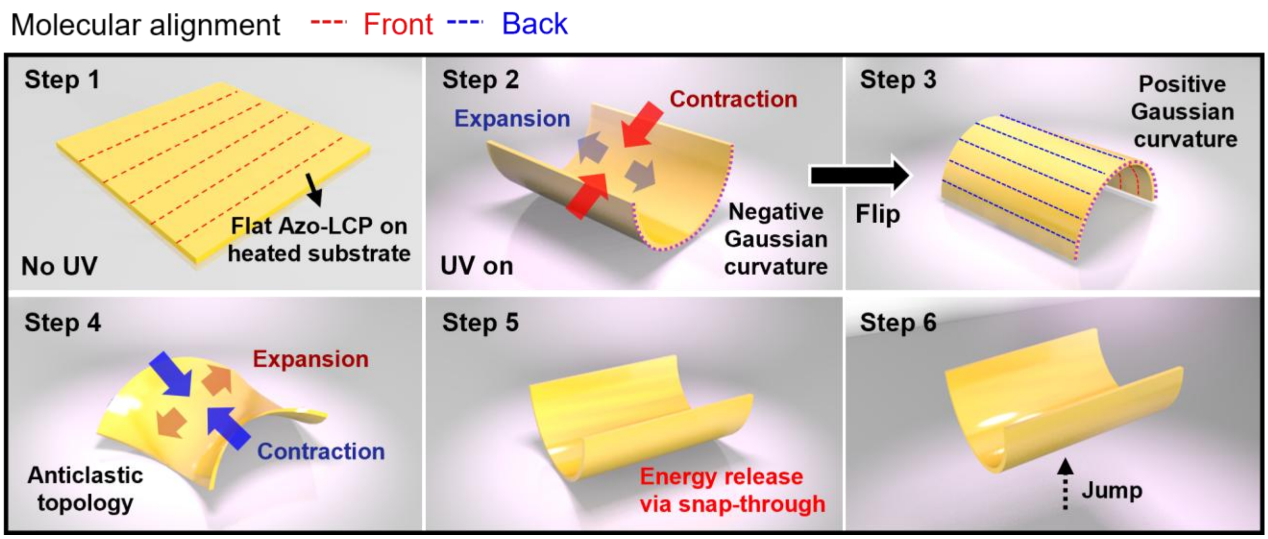

Fig. S2. Three-dimensional scheme illustrating the topological transition of Azo-LCP upon UV irradiation.

Photomechanical jumping mechanism

Azobenzene is covalently embedded in LCP, and the UV light triggers trans-cis photoisomerization, generating photogenerated stress through mechanical pulling of adjacent molecules, thereby inducing an order-disorder transition of LC molecules (*48*). The resulting shape of Azo-LCP was engineered to form a saddle-like anticlastic structure having both positive and negative Gaussian curvature, representing a mechanically unstable, high-energy intermediate state in the snap-through process (*49*). This configuration was achieved by encoding the 270° super twisted nematic (STN) structure within the Azo-LCP. For photo-mechanical jumping via snap-through, Azo-LCP was first placed on a heating plate and UV light irradiation was followed as illustrated in Fig. S2. Upon UV light irradiation, the Azo-LCP film reconfigured from a flat 2D film shape into a monoclastic structure with a single negative Gaussian curvature, reaching an equilibrium state of the snap-through process. Due to the exponential decay of UV light intensity described by the Beer-Lambert law, the number of activated azobenzene molecules also exponentially decreases along the thickness of the film, resulting in a directional bending toward

the UV-exposed side (*50*). This phenomenon is referred to as the skin-bulk effect. Subsequently, when the structure is inverted to attain positive Gaussian curvature which is bistable with the initial structure, shape-reconfiguration toward a saddle-like anticlastic structure begins, driven by the accumulation of photogenerated stress. As the Azo-LCP exhibits parallel contraction and perpendicular expansion to the alignment direction, the direction of expansion and contraction is orthogonally generated between the bottom and top surfaces due to the 270° STN alignment. During the transition of a monoclastic structure with positive Gaussian curvature to an anticlastic structure, gradual deformation leads to the accumulation of photogenerated stress concentrated in the central region of the film. Subsequently, when the accumulated stress exceeds the energy barrier of snap-through, a rapid topological transition occurs, resulting in a monoclastic structure with negative Gaussian curvature. This sudden release of accumulated stress causes the Azo-LCP to jump, as the central region strongly impacts the ground.

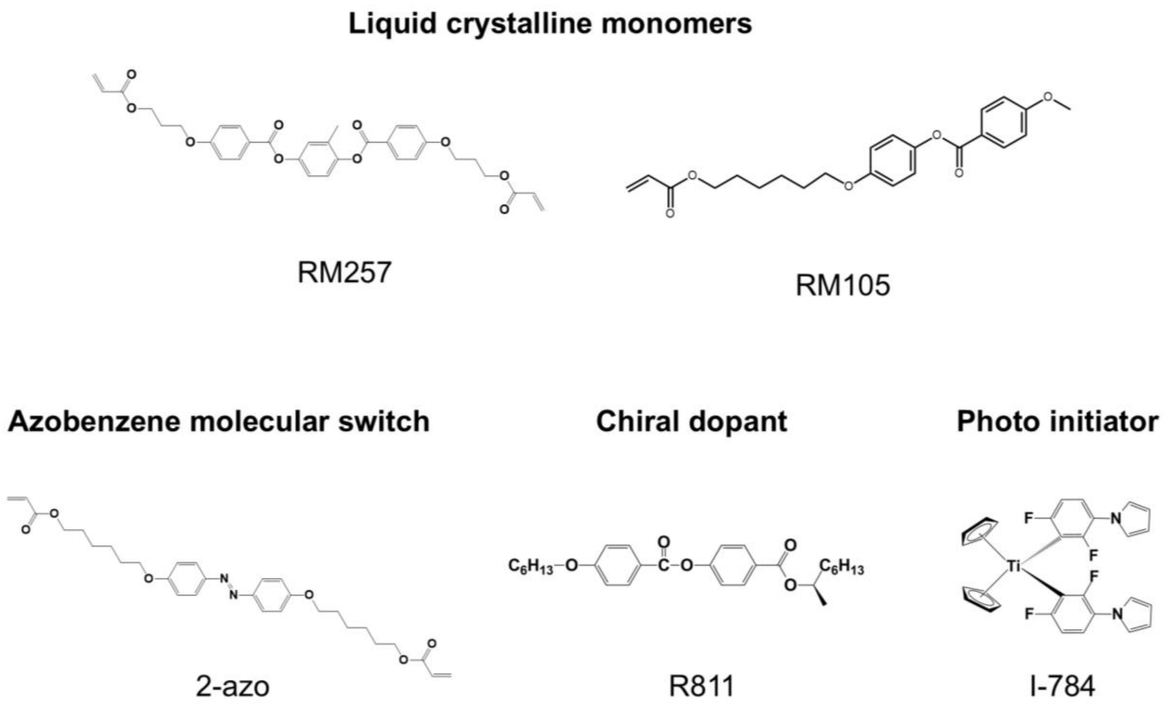

Fig. S3. Chemical structures of components forming Azo-LCP soft robots.

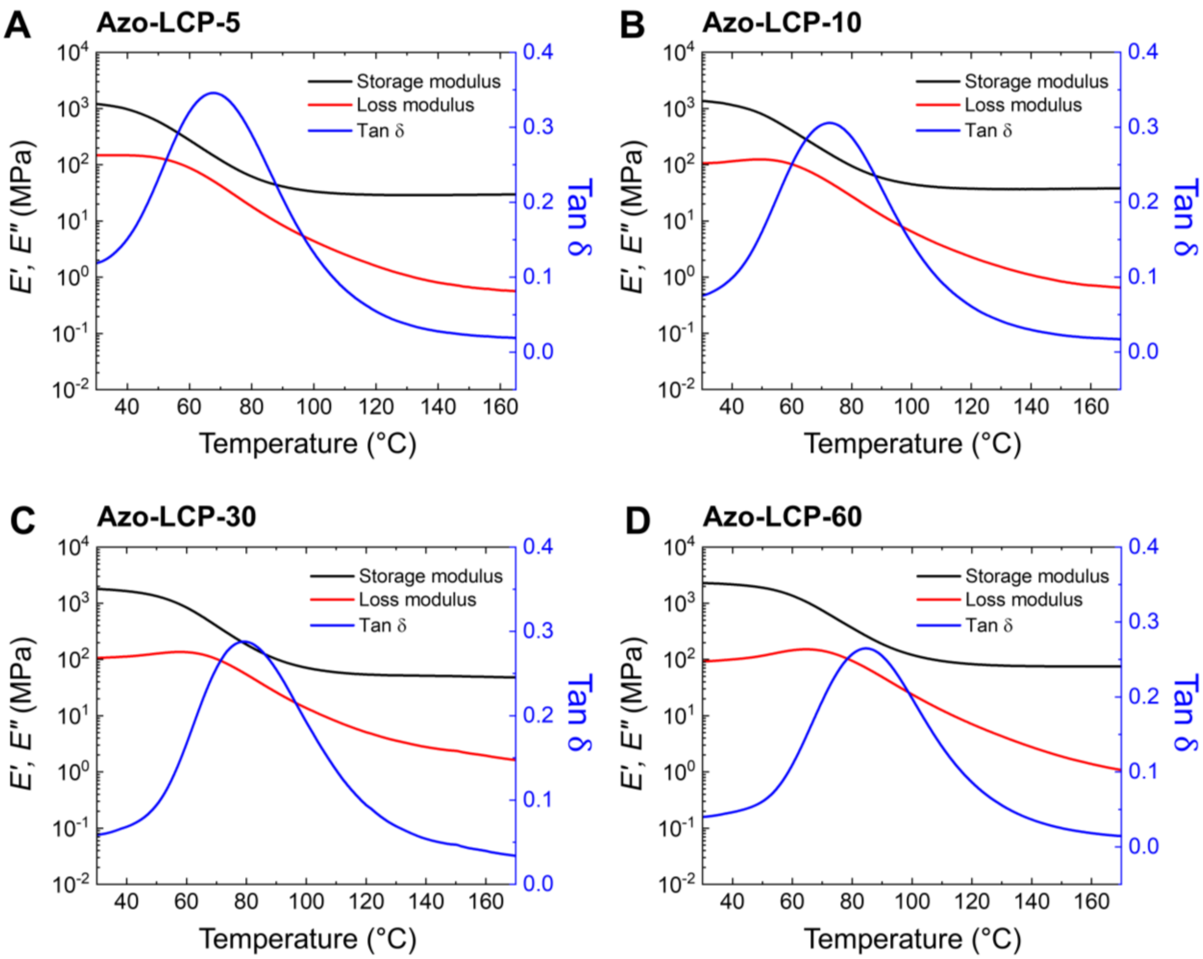

Fig. S4. Thermomechanical response of Azo-LCPs. (A–D) Thermomechanical properties of the uniformly crosslinked Azo-LCPs obtained by dynamic mechanical analysis (DMA). Black lines correspond to storage modulus (E′), red lines indicate loss modulus (E″), and blue lines are equivalent to tan δ of the Azo-LCPs.

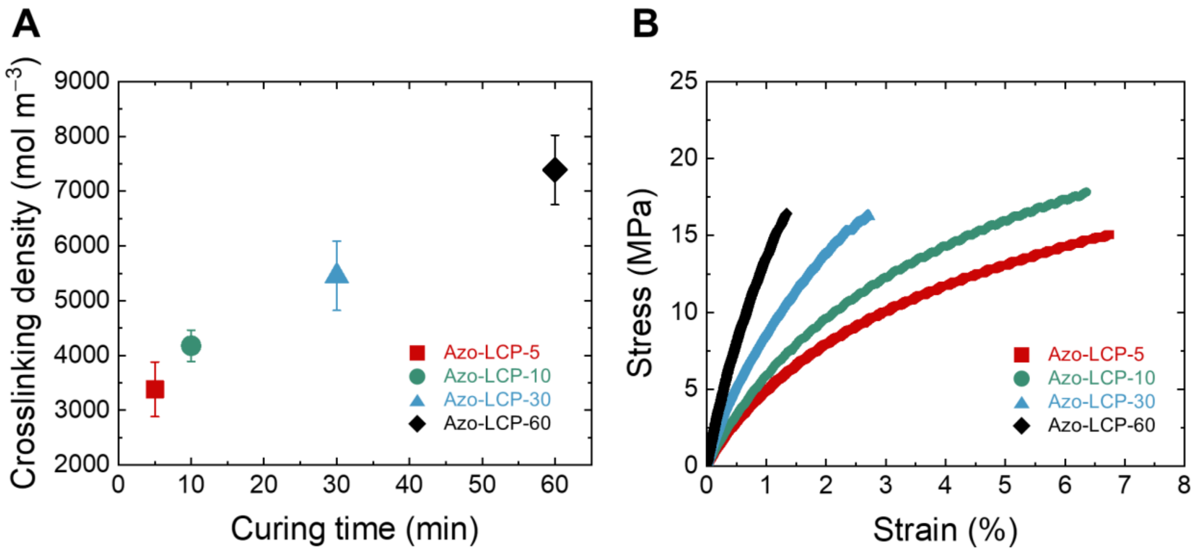

Fig. S5. Material properties of uniformly crosslinked Azo-LCPs. (A) Crosslinking density of Azo-LCPs prepared with different curing times, calculated using Flory's rubber elasticity theory. (B) Stress–strain curves of Azo-LCPs with different crosslinking densities.

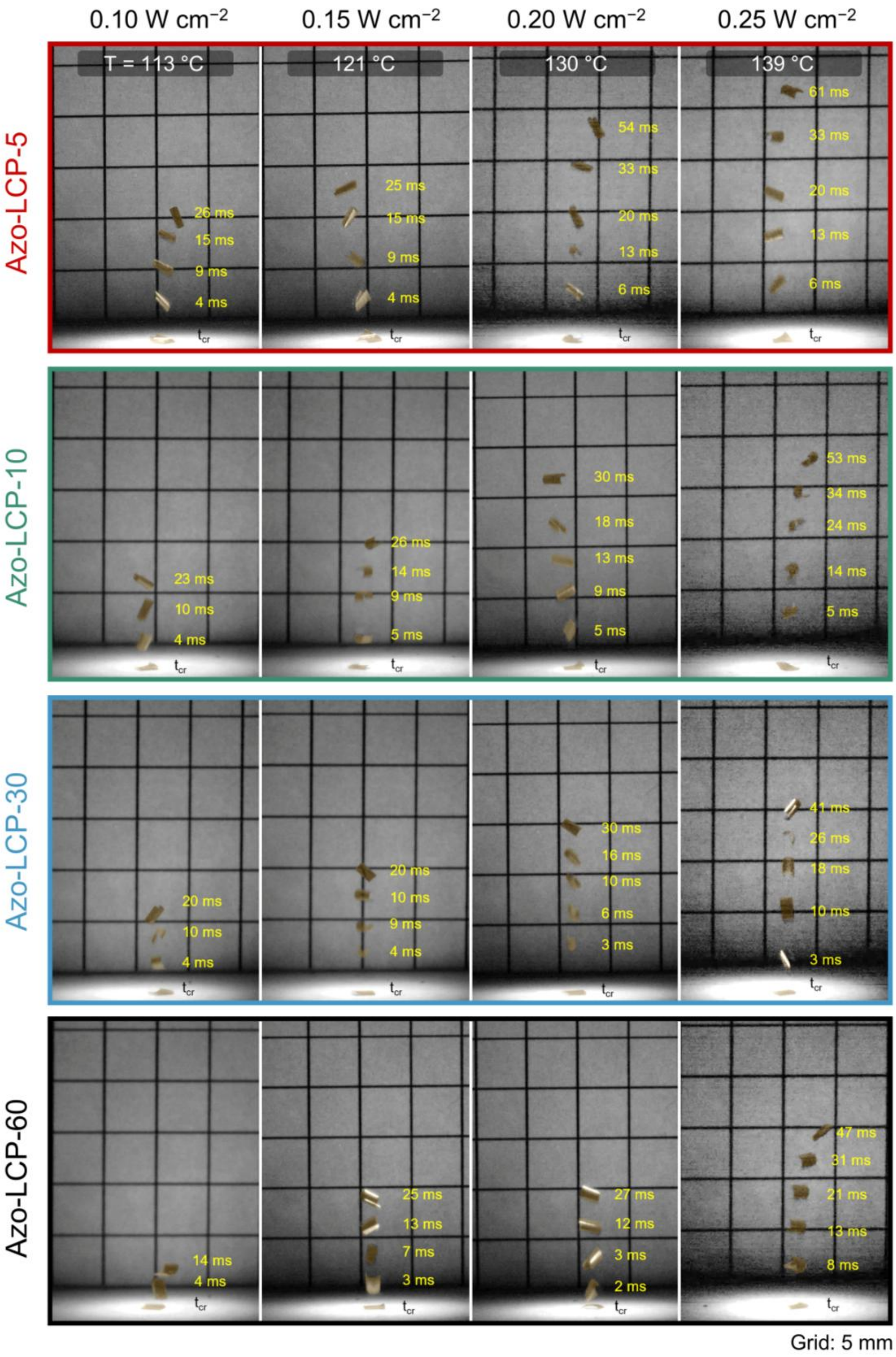

Fig. S6. Jumping behavior of Azo-LCPs with varying crosslinking densities under different UV light intensity (Substrate temperature = 120 ºC).

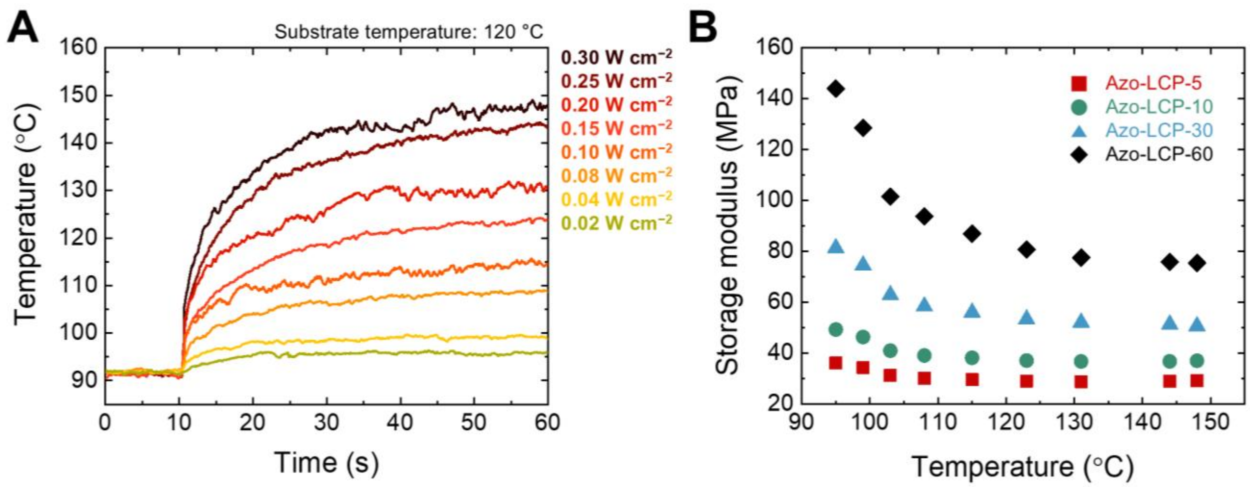

Fig. S7. Temperature profiles under UV irradiation and their correlation with storage modulus. (A) Time-resolved temperature profiles of the Azo-LCP under UV-light irradiation at various intensities ranging from 0.02 to 0.30 W cm^{-2}, measured using a thermal imaging camera. (B) Correlation between saturated body temperature and storage modulus of Azo-LCPs with different crosslinking densities.

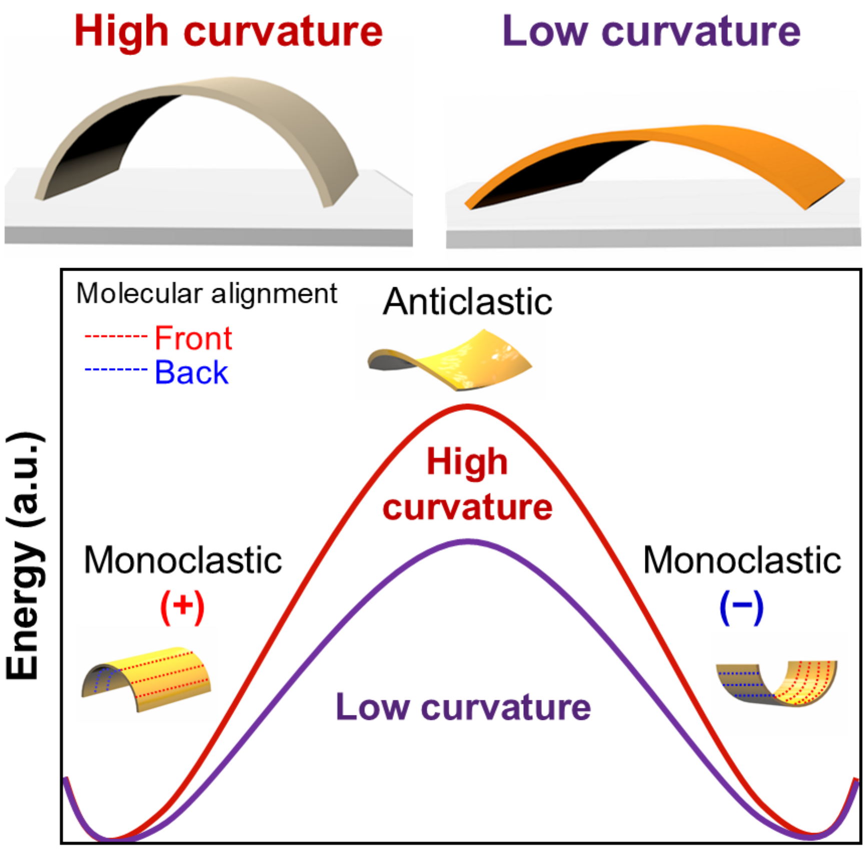

Fig. S8. The potential energy diagram of Azo-LCPs with high and low curvatures.

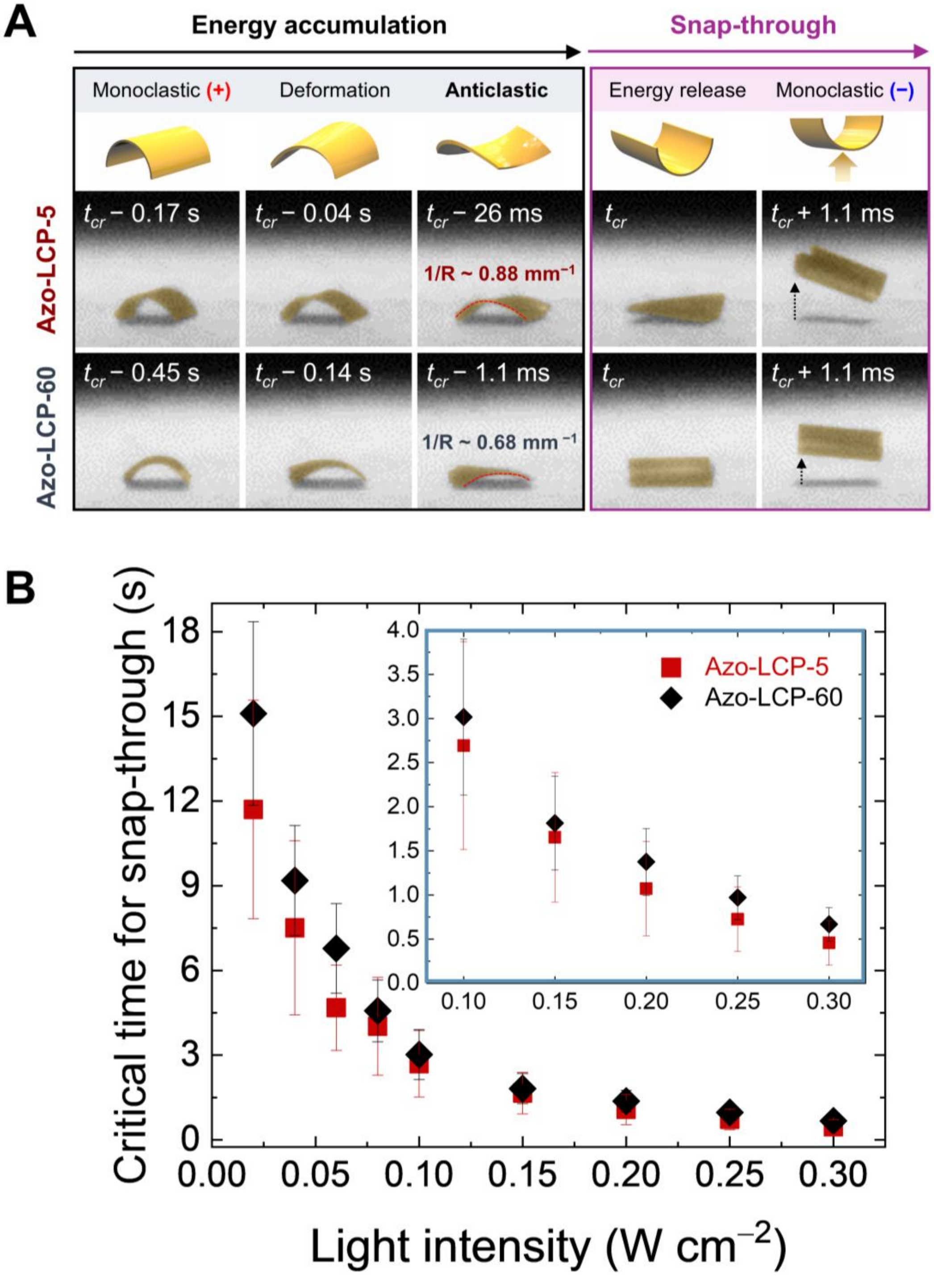

Fig. S9. Topological transitions and critical snap-through times. (A) Photographs and schematic illustrations comparing the photo-induced topological transitions of Azo-LCP-5 and Azo-LCP-60 under identical actuation conditions (Body temperature = 148 ºC, UV-light intensity = 0.3 W cm^{-2}). (B) Critical time for snap-through (t_{cr}) of Azo-LCP-5 and Azo-LCP-60 as a function of light intensity.

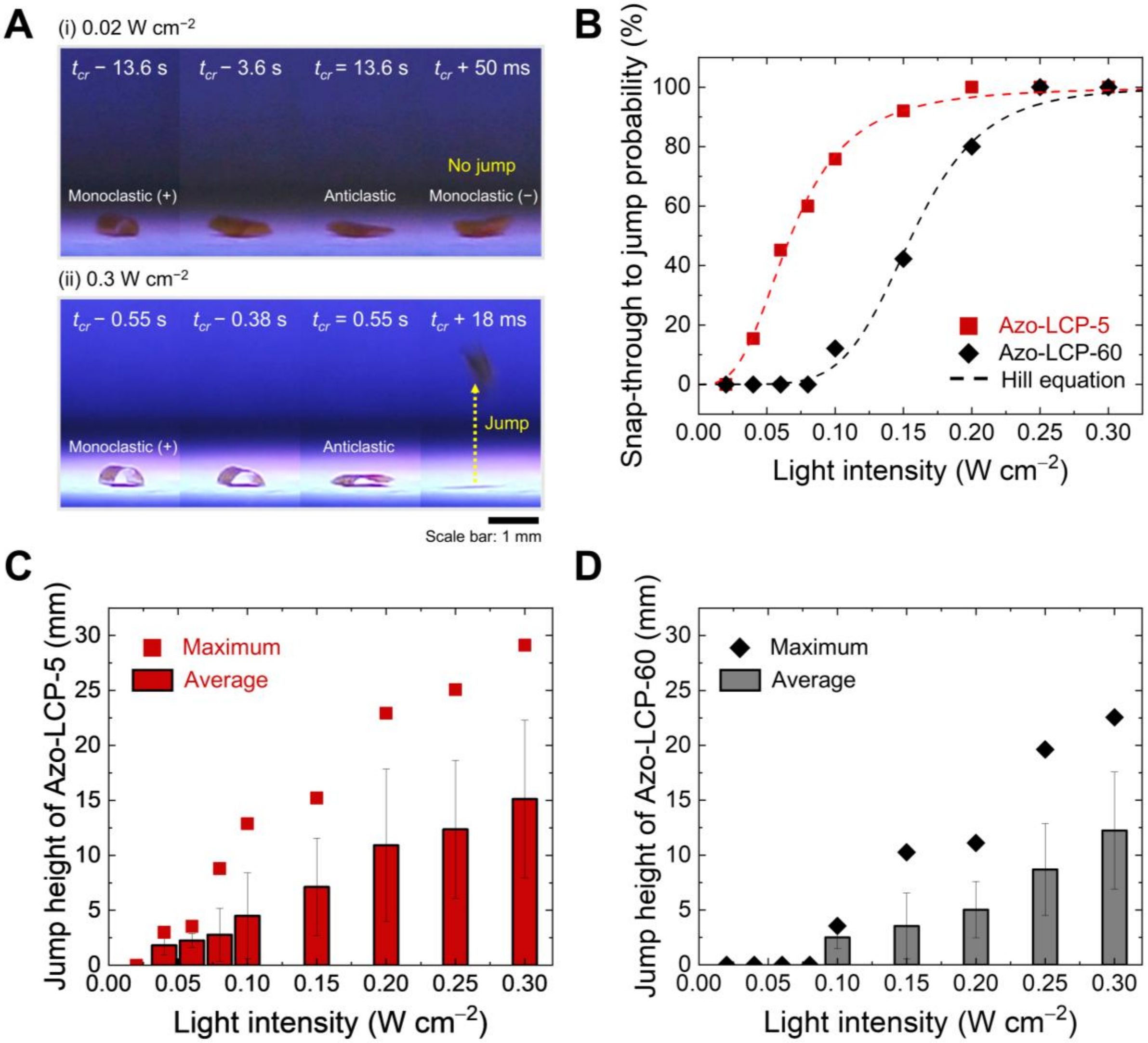

Fig. S10. Snap-through-to-jump transitions and jumping performance of uniformly crosslinked Azo-LCPs. (A) Digital images demonstrating snap-through and jumping of Azo-LCP-5 under varied UV light intensities of (i) 0.02 W cm^{-2} and (ii) 0.3 W cm^{-2}. (B) Snap-through-to-jump probability of Azo-LCP-5 and Azo-LCP-60 at various light intensities. (C-D) Average and maximum jump heights of Azo-LCP-5 and Azo-LCP-60 demonstrated as a function of light intensities.

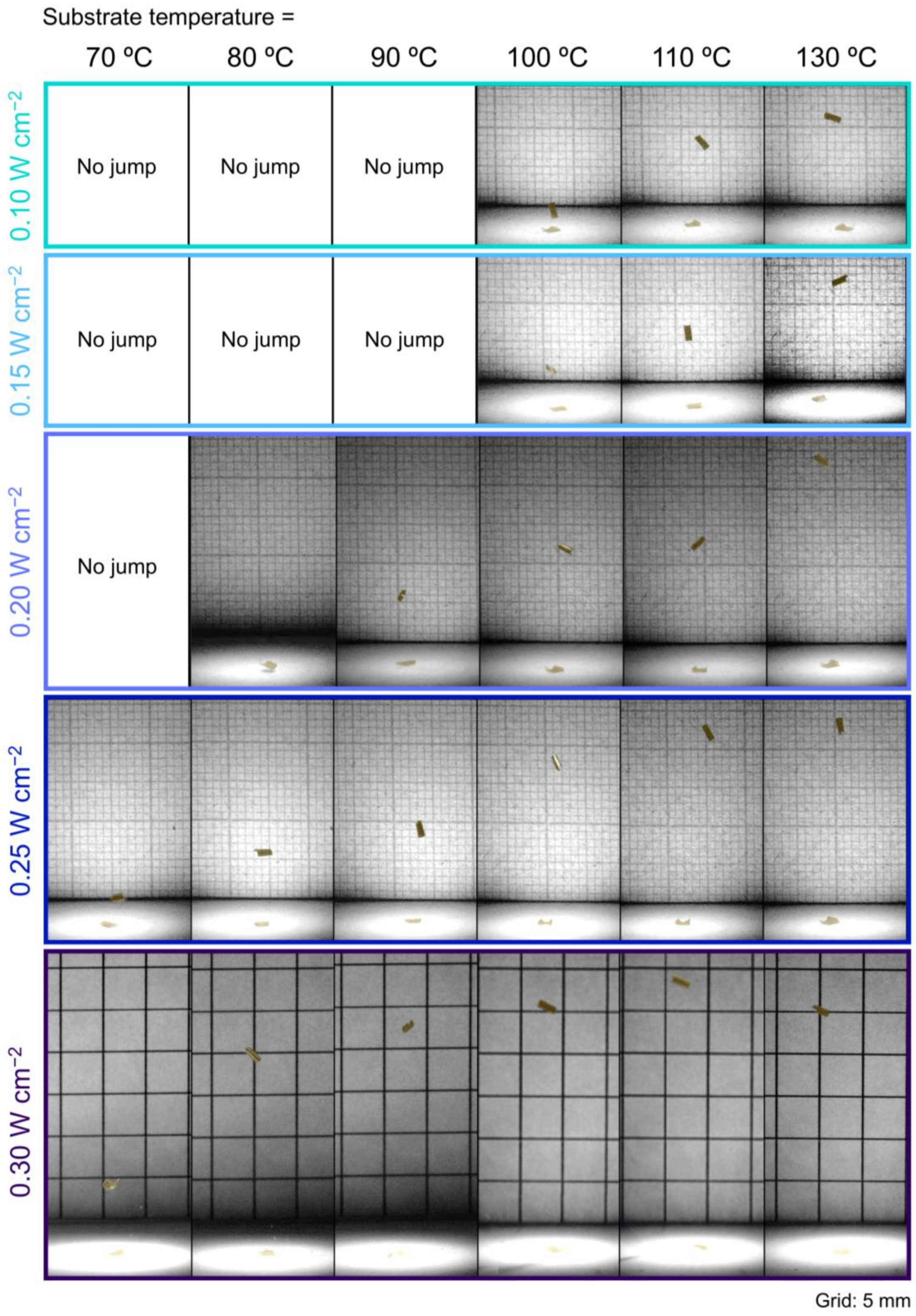

Fig. S11. Jumping behaviors of Azo-LCP-5 under varied UV light intensities and substrate temperature.

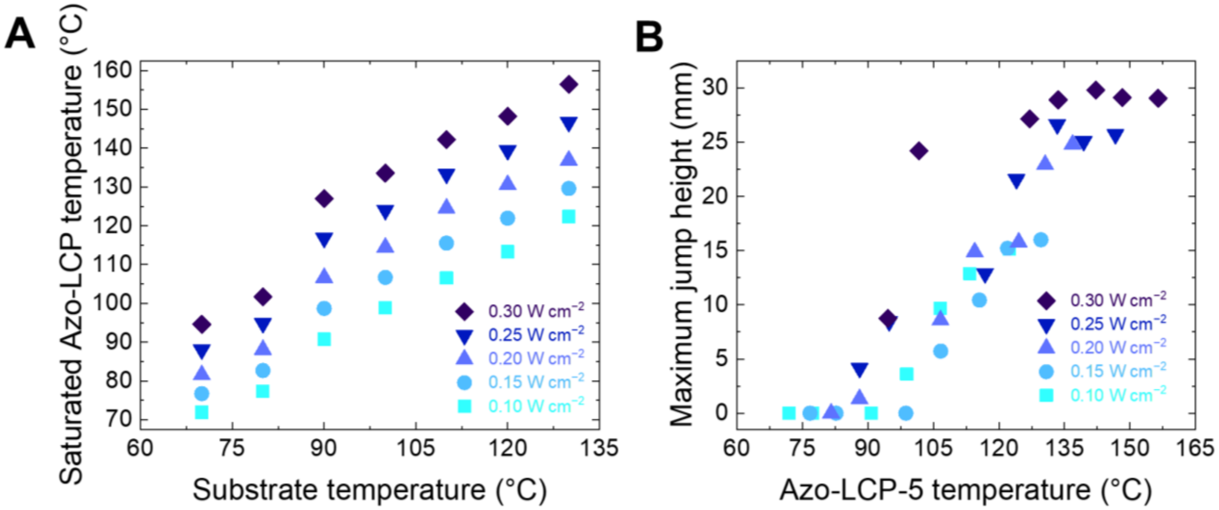

Fig. S12. Azo-LCP temperature and jump performance. (A) Saturated Azo-LCP temperature and (B) maximum jump height of Azo-LCP-5 depending on UV-light intensities and substrate temperatures.

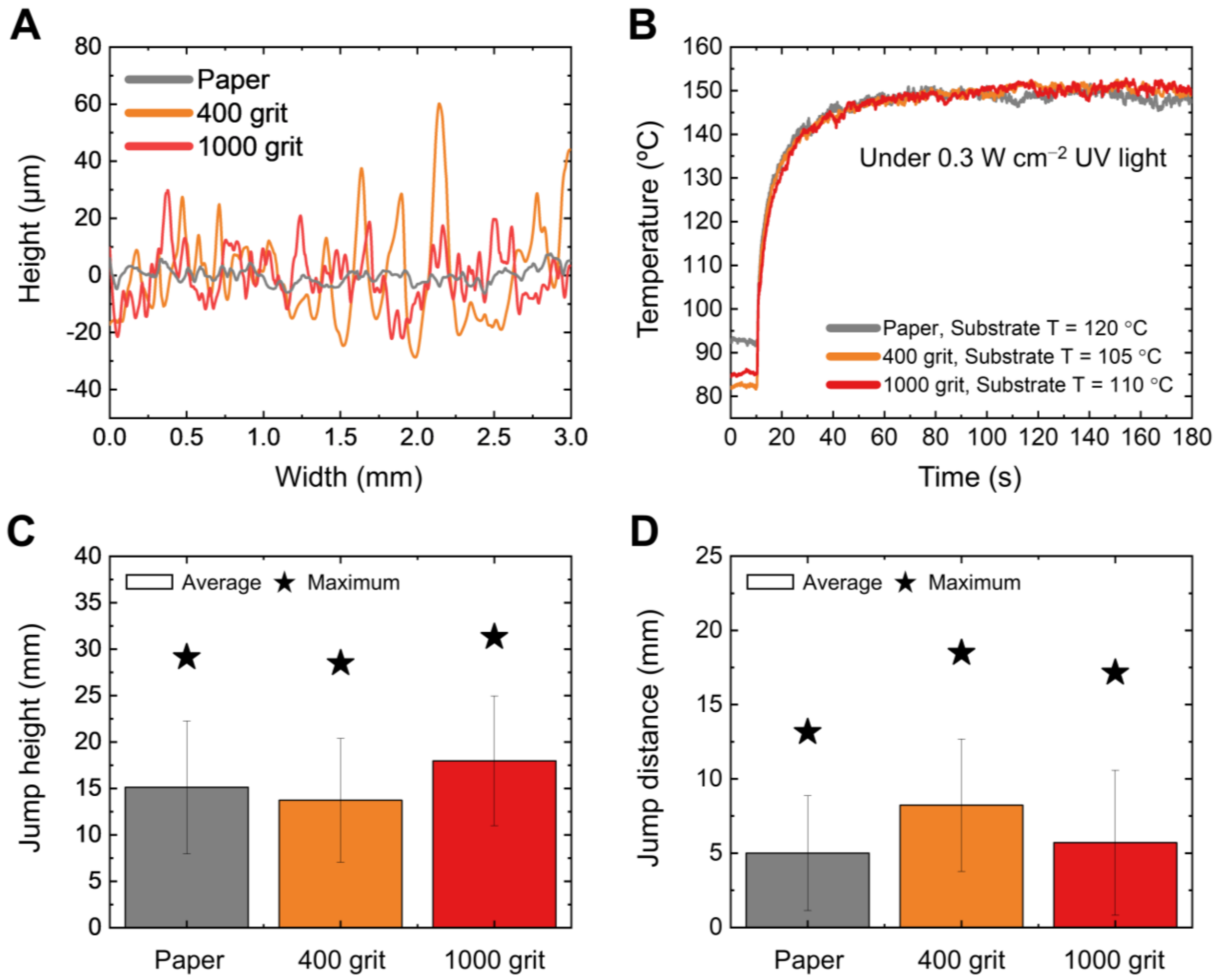

Fig. S13. Substrate-dependent photomechanical response and jump performance. (A) Surface height profiles of an originally used white paper substrate and sandpapers with grit sizes of 400 and 1000. (B) Temperature variation of the paper and sandpapers under 0.3 W cm^{-2} UV irradiation at different controlled substrate heating temperatures. Average and maximum photomechanical (C) jump height and (D) jump distance measured on different substrates.

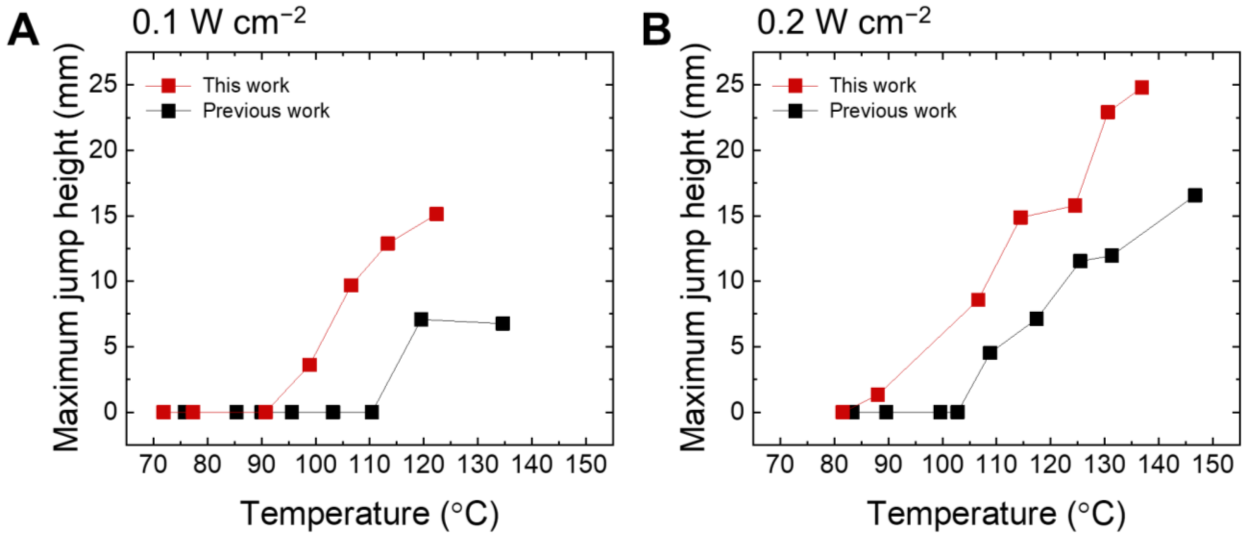

Fig. S14. Maximum jump height comparisons. (A-B) Maximum jump height comparison of Azo-LCP-5 and a previously reported photomechanical Azo-LCP under different light intensities and temperatures.

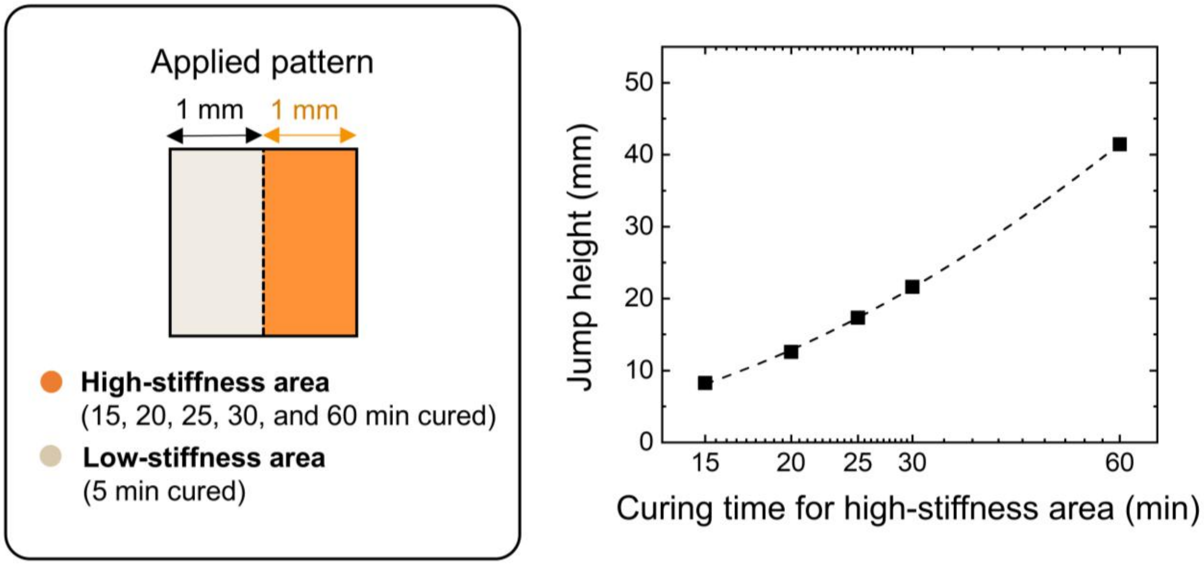

Fig. S15. Jump height comparisons of asymmetrically patterned Azo-LCPs with high-stiffness areas subjected to different curing times.

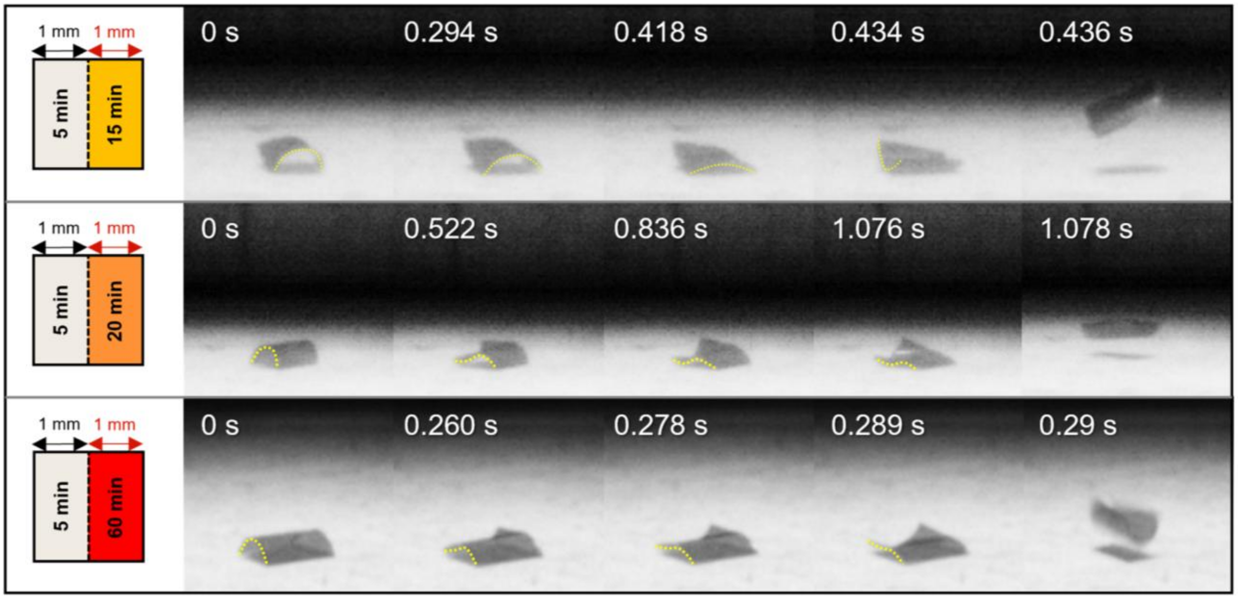

Fig. S16. Topological transition digital images of asymmetrically patterned Azo-LCPs with 5 min cured low-stiffness area and 15 min, 20 min, and 60 min cured high-stiffness areas.

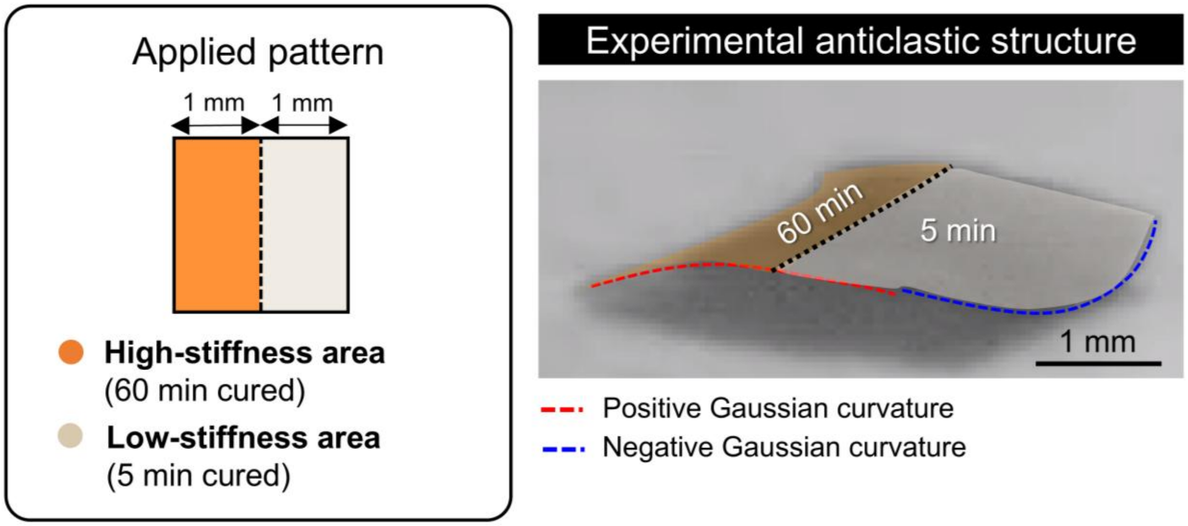

Fig. S17. Experimental anticlastic structure of asymmetrically patterned Azo-LCP upon UV irradiation of 0.3 W cm^{-2} and substrate temperature of 120 °C.

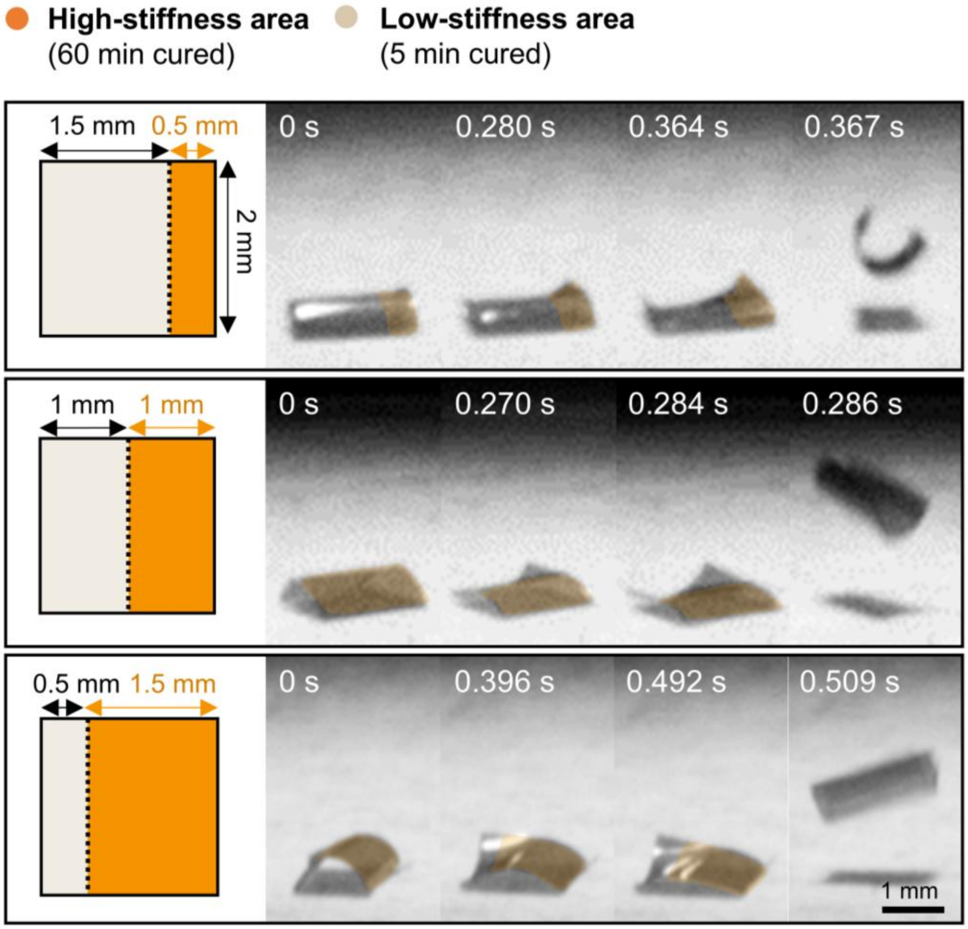

Fig. S18. Digital images showing the topological transition of asymmetrically patterned Azo-LCPs with varying ratios of soft and rigid regions.

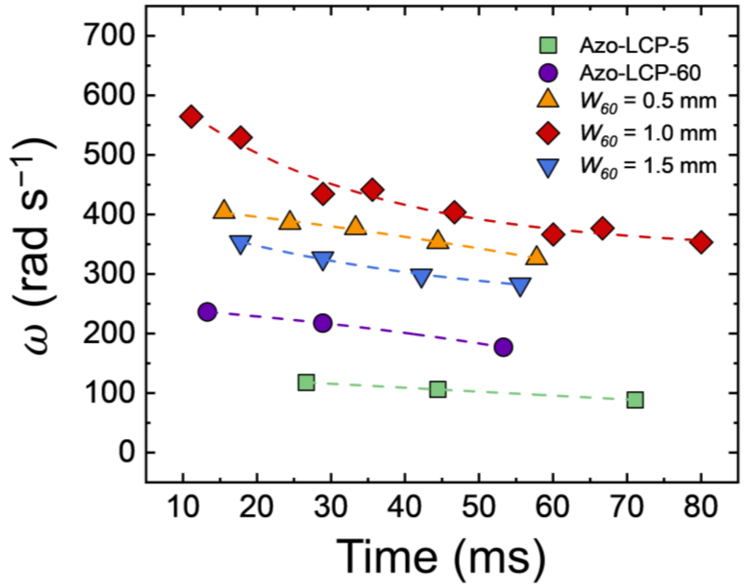

Fig. S19. Calculated angular velocities of Azo-LCP-5, Azo-LCP-60, and asymmetrically patterned Azo-LCPs according to time.

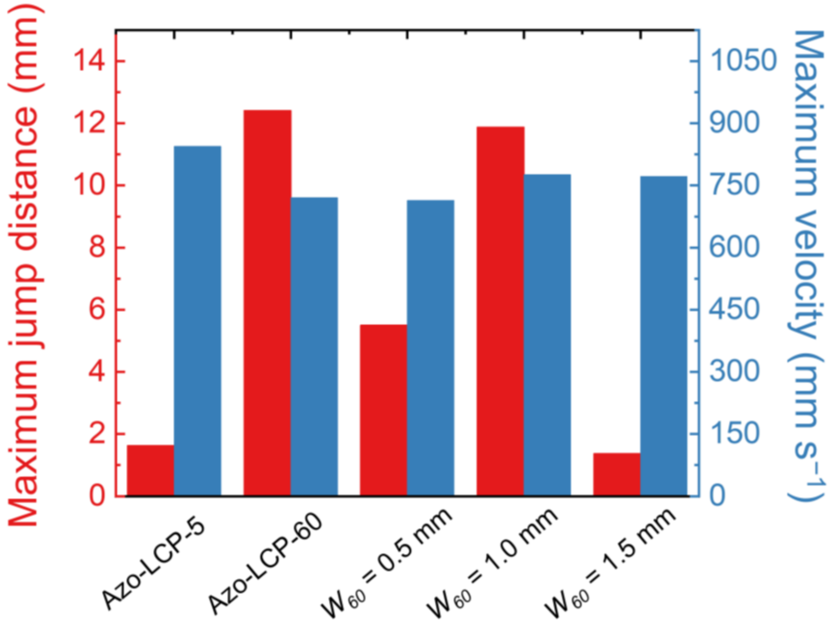

Fig. S20. Comparison of measured maximum jump distance and velocity achieved by Azo-LCPs.

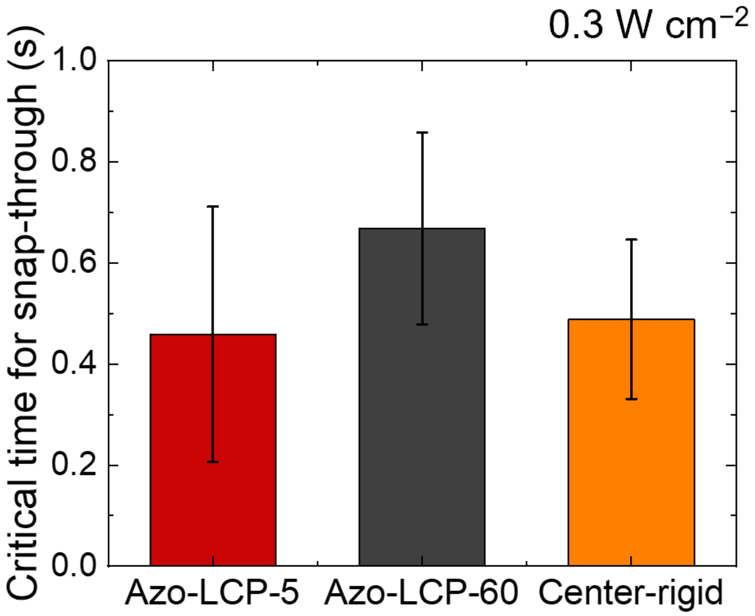

Fig. S21. Comparison of critical time for snap-through under 0.3 W cm^{-2} UV light among uniformly crosslinked Azo-LCPs (Azo-LCP-5 and Azo-LCP-60) and the center-rigid Azo-LCP (D_{60} = 1 mm).

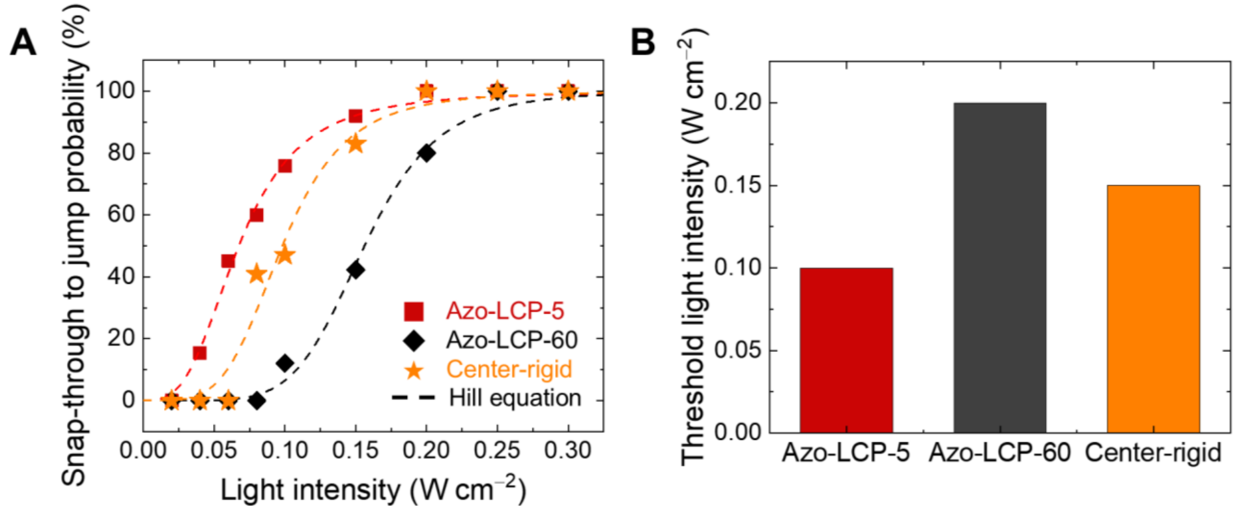

Fig. S22. Jump probability and threshold light intensity of Azo-LCPs. (A) Snap-through to jump probability of Azo-LCPs as a function of light intensities. (B) The threshold light intensity required for Azo-LCPs to achieve a jump probability above 75%.

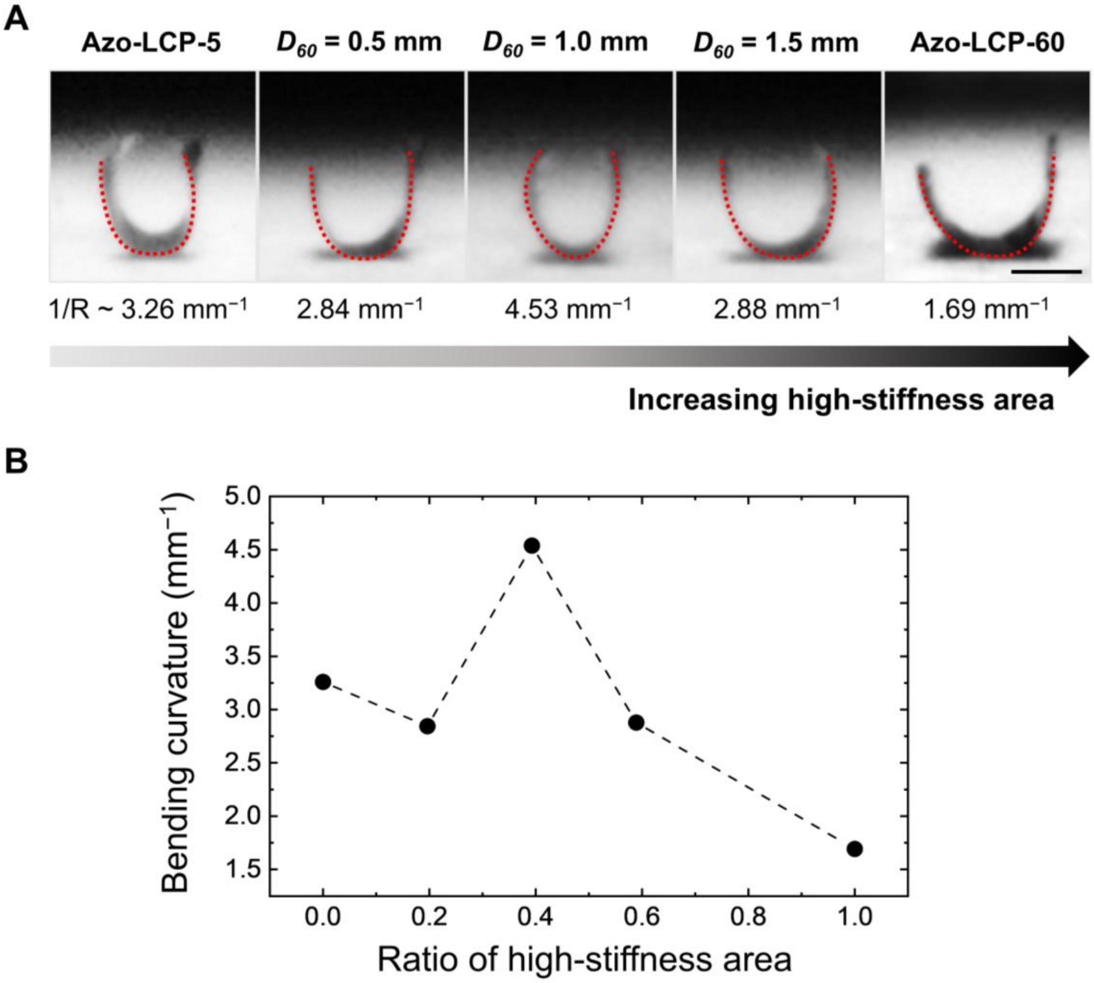

Fig. S23. UV-induced bending of Azo-LCPs. (A) Photographs of UV-induced bending and (B) the measured curvature values for uniformly crosslinked Azo-LCPs (Azo-LCP-5 and Azo-LCP-60) and center-rigid Azo-LCPs (D_{60} = 0.5 mm, D_{60} = 1.0 mm, and D_{60} = 1.5 mm). Scale bar: 0.5 mm.

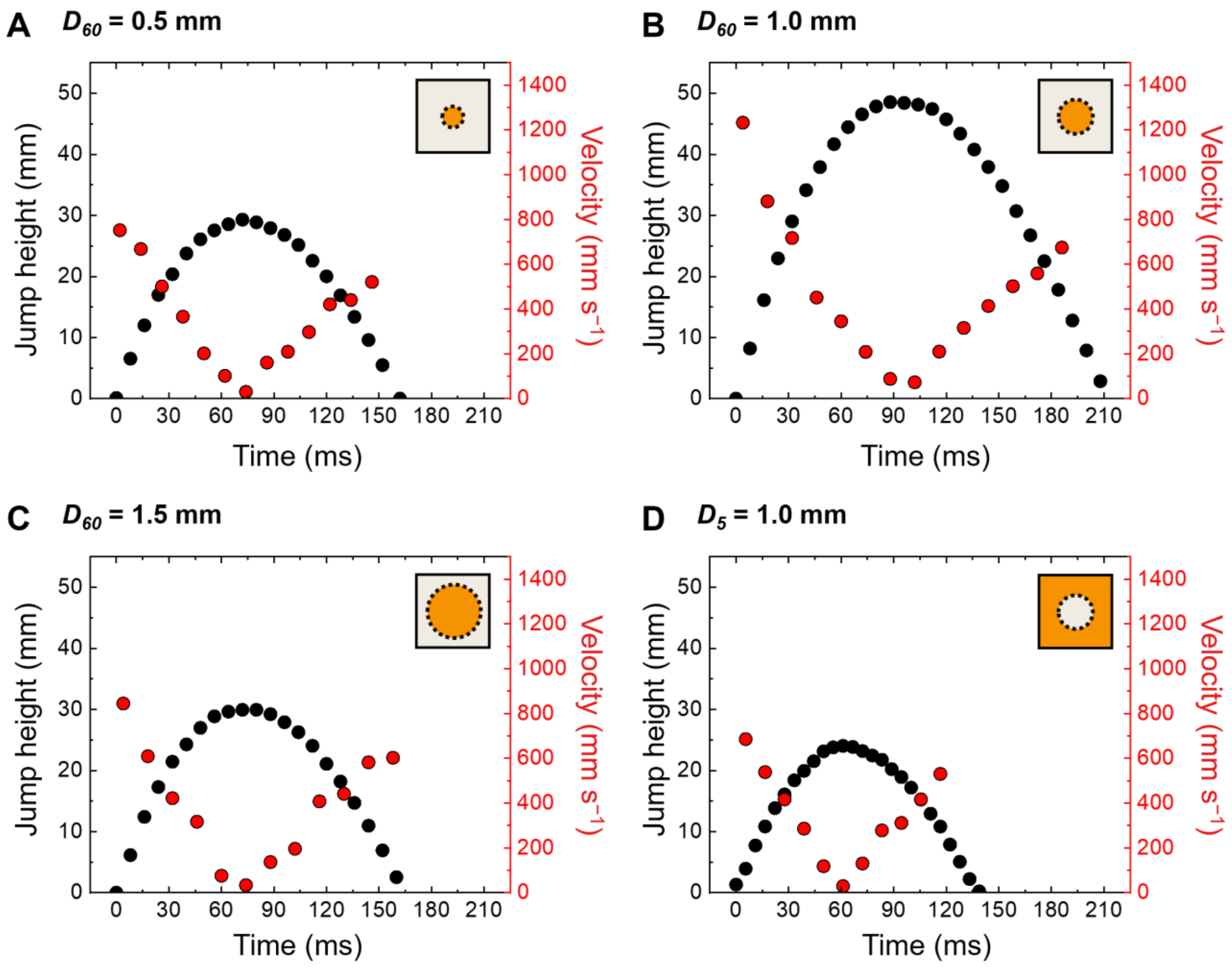

Fig. S24. Measured jump height and velocity achieved by center-rigid and inversely patterned (D_5 = 1.0 mm) Azo-LCPs according to time (A–D).

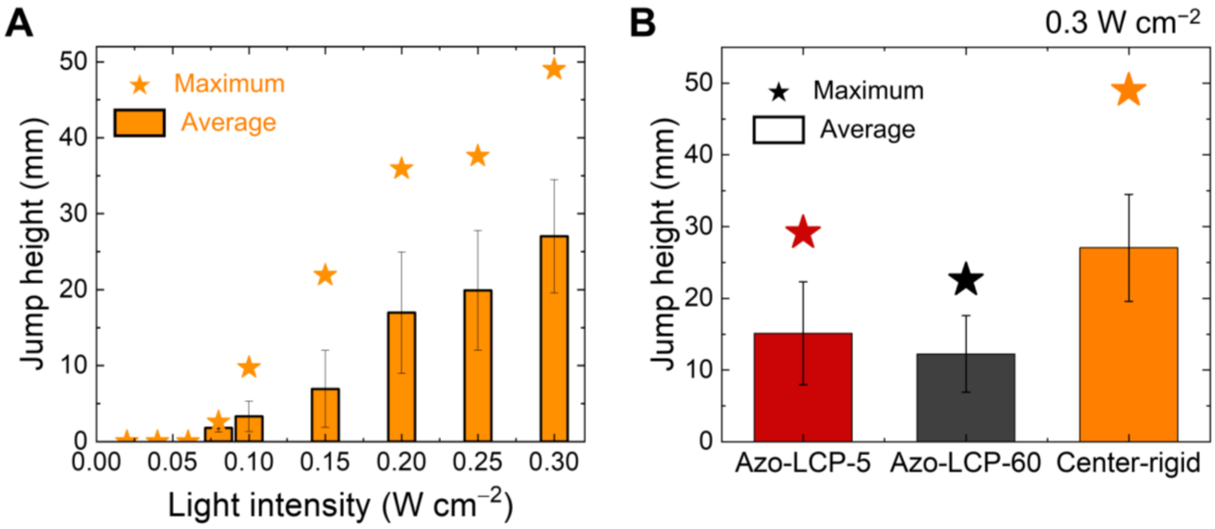

Fig. S25. Maximum and average jump heights. (A) Maximum and average jump heights of center-rigid Azo-LCP at varied light intensities. (B) Comparison of maximum and average jump heights among uniformly crosslinked Azo-LCPs and center-rigid Azo-LCP (D_{60} = 1.0 mm).

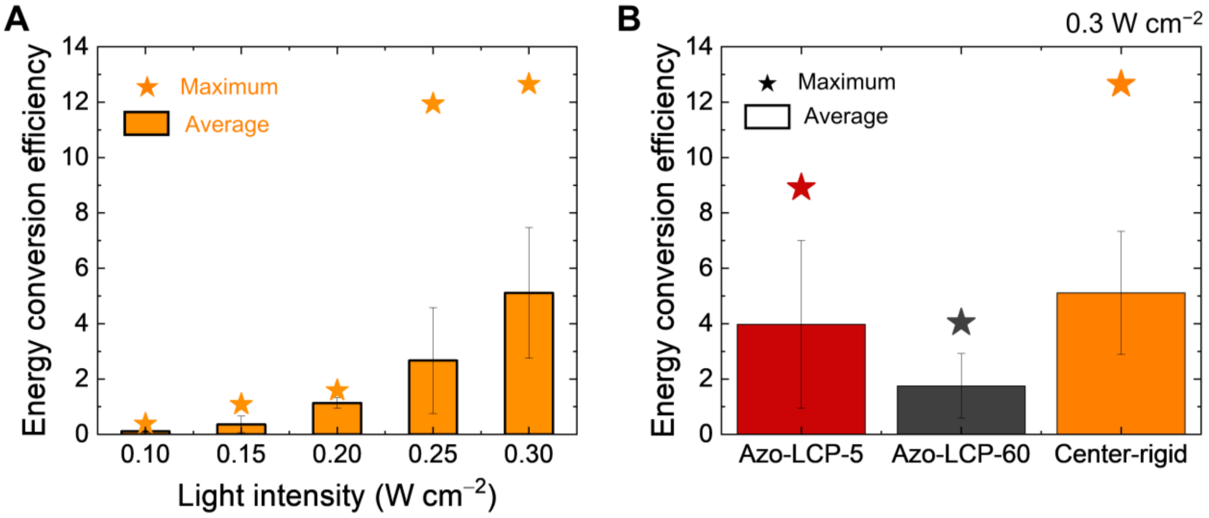

Fig. S26. Calculated energy conversion efficiency of jumping. (A) Energy conversion efficiency from incident light energy to gravitational potential energy for the center-rigid Azo-LCP under varying UV light intensities. (B) Comparison of the energy conversion efficiency among uniformly crosslinked Azo-LCPs and center-rigid Azo-LCP (D_{60} = 1.0 mm) under a UV light intensity of 0.3 W cm^{-2}.

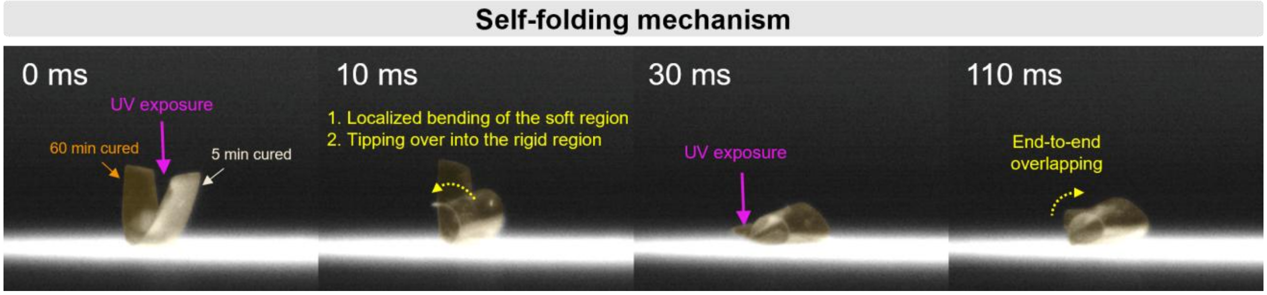

Fig. S27. Long axis bending (corresponding to Mode I deformation) of the dual-mode Azo-LCP jumper with an aspect ratio of 2.

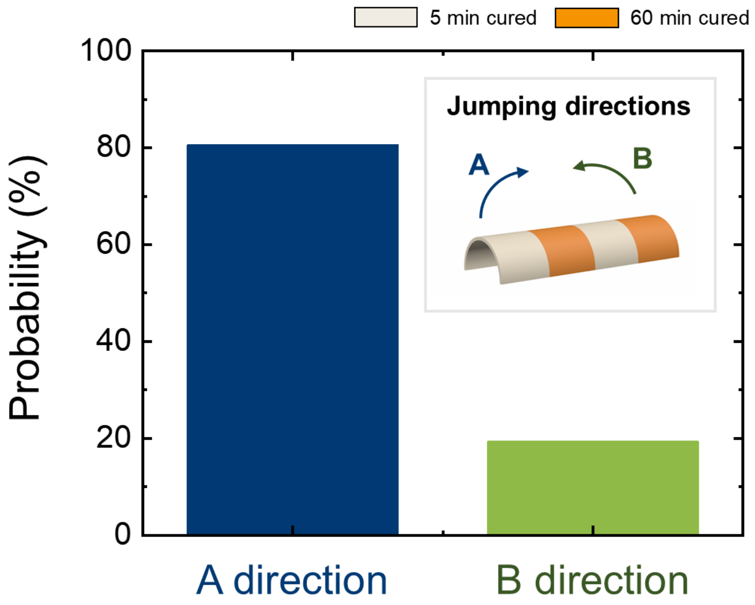

Fig. S28. The probability of the directional jump from soft to rigid region (A direction), and from rigid to soft region (B direction).

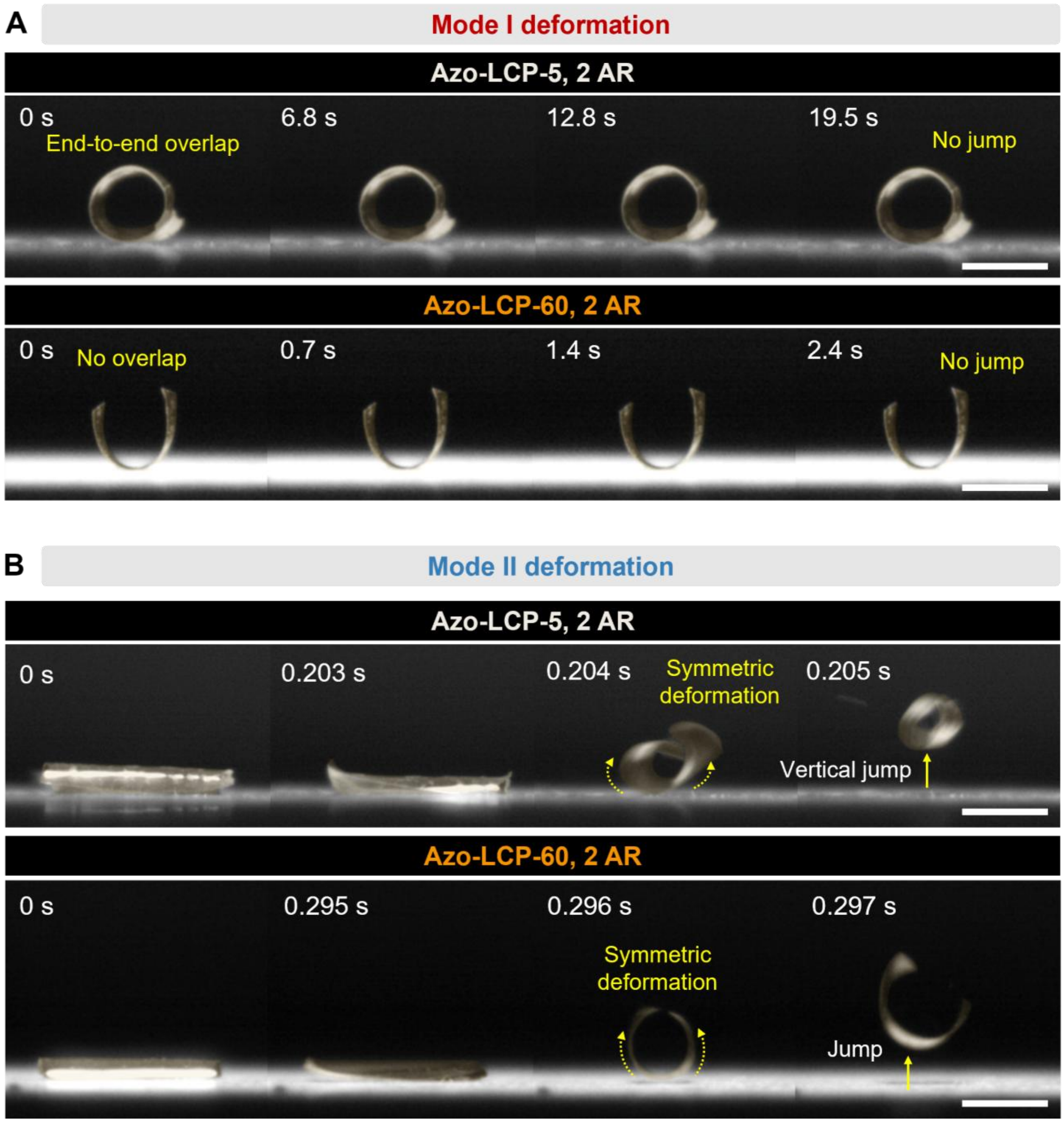

Fig. S29. Mode I and Mode II deformations of high-aspect-ratio Azo-LCPs. (A) Mode I and (B) Mode II deformation of uniformly crosslinked Azo-LCPs with an aspect ratio of 2, along with the corresponding jumps. Scale bars: 2 mm.

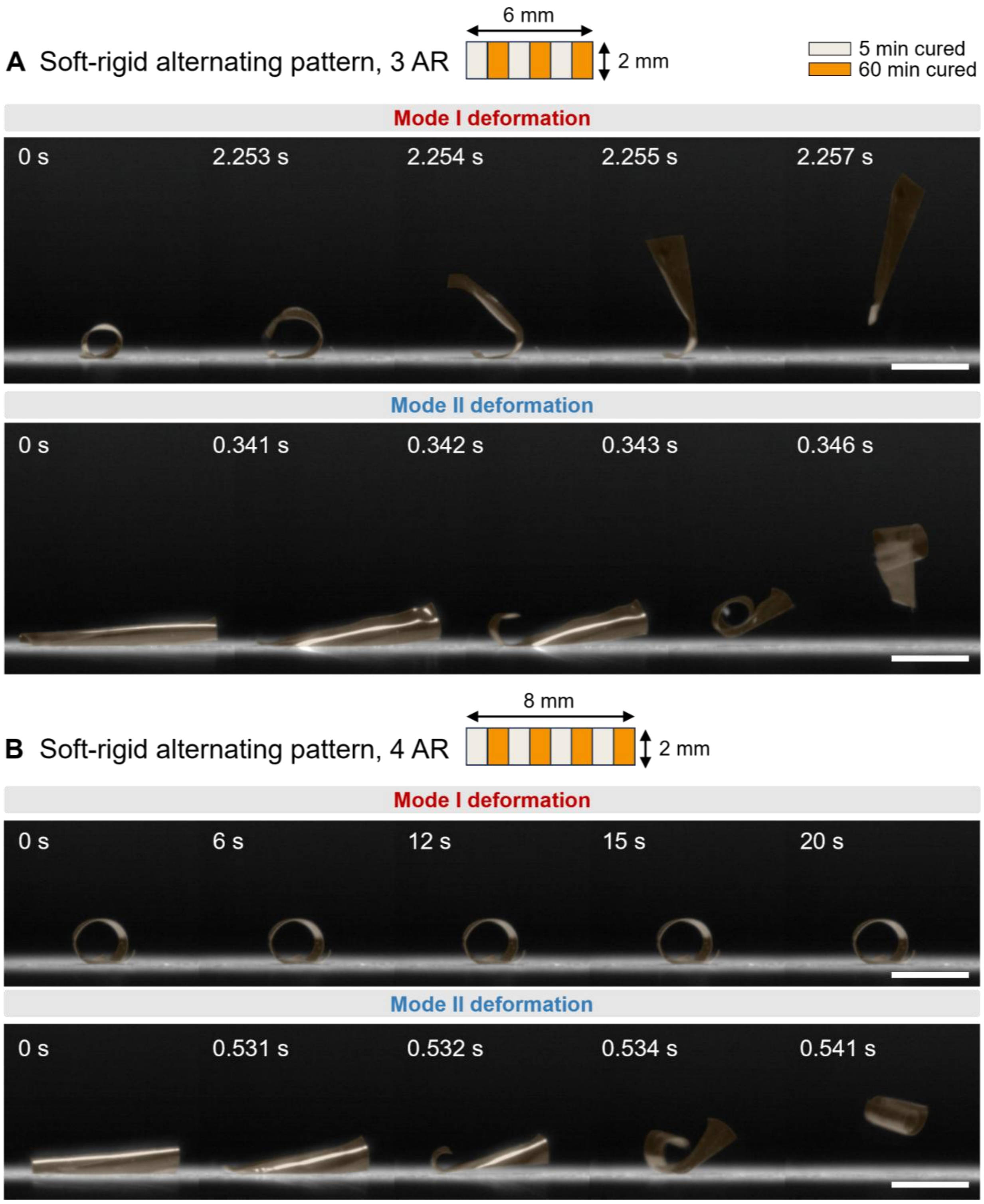

Fig. S30. Mode I and Mode II deformations of high-aspect-ratio Azo-LCPs with a soft-rigid alternating pattern. (A) Mode I and Mode II deformations and the resulting jumps of Azo-LCPs with the soft-rigid alternating pattern and an aspect ratio of 3. (B) Corresponding deformation and jumping behavior observed in samples with an aspect ratio of 4. Scale bars: 2 mm.

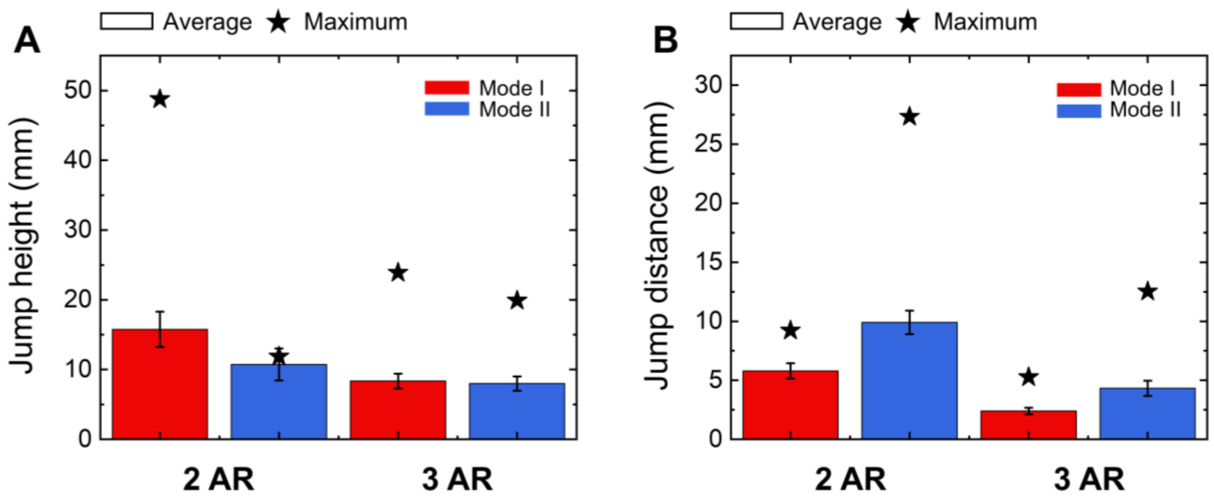

Fig. S31. Comparison of the jump height and distance for (A) Mode I and (B) Mode II jumps of dual-mode Azo-LCPs with a soft-rigid alternating pattern, having aspect ratios of 2 and 3.

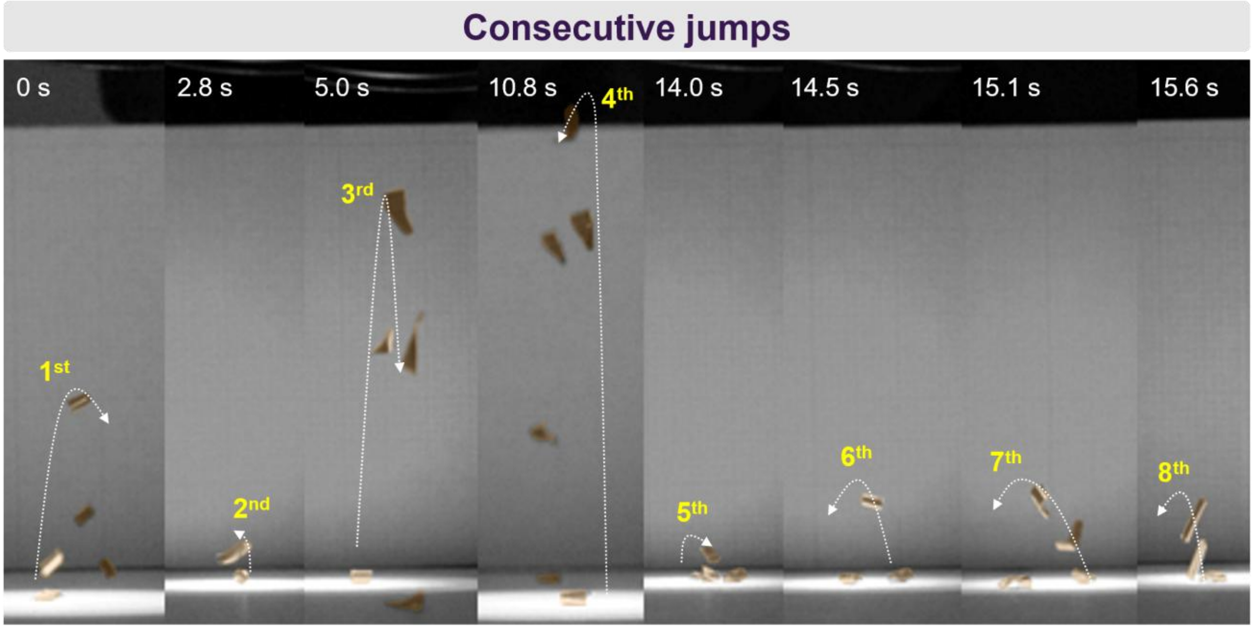

Fig. S32. Consecutive jumps of a dual-mode Azo-LCP jumper with an aspect ratio of 2.

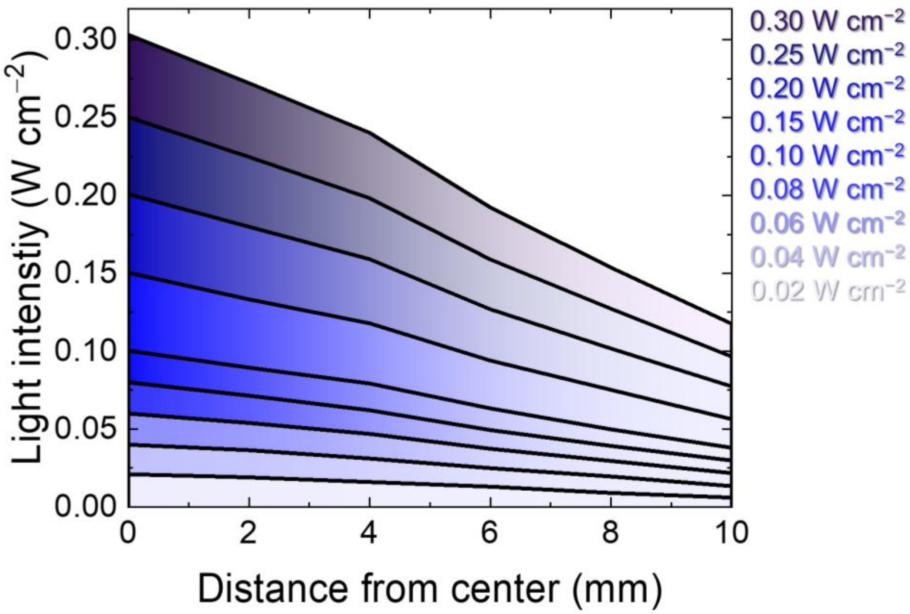

Fig. S33. Light intensity profile as a function of distance from the beam center.

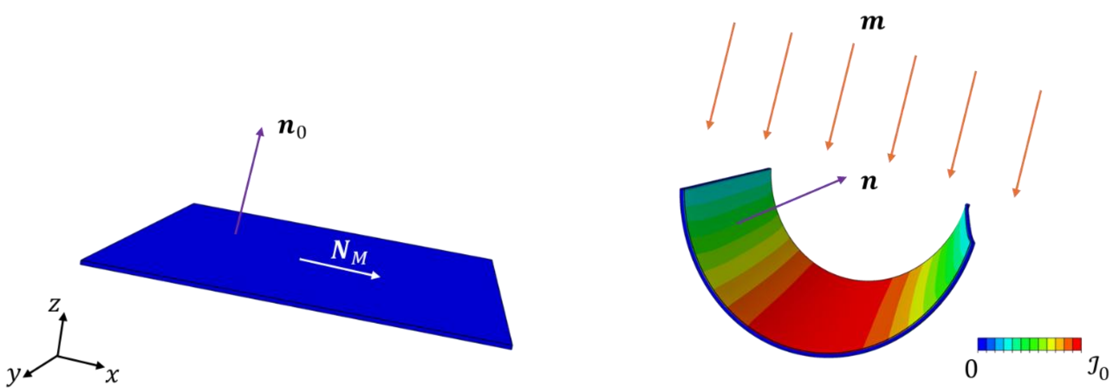

Fig. S34. The illustration of deformation-dependent light intensity. $\boldsymbol{N}_{\mathrm{M}}$ is the direction of the mesogens, $\boldsymbol{m}$ is the light direction**,** $\boldsymbol{n}_0$ and $\boldsymbol{n}$ are the initial and current surface normal vector, respectively. The color shows the effective light intensity.

Modeling and simulations

To reveal the underlining mechanism of the Azo-LCP jumpers, we employ a photomechanical continuum model to simulate the deformed configuration and snap-through instability of the spatially patterned stiffness variation (37, 38). The model focus on the photogenerated strain to drive the deformation, which, in turn, modifies the process of *trans–cis* photoisomerization. However, it should be noted that the photomechanical coupling of the Azo-LCP is highly nonlinear and determined by the temperature. To simplify the model, we neglect the temperature change during the deformation process and only focus on the anisotropic active photogenerated strain while using an isotropic neo-Hookean solid model. Azobenzene will generate an anisotropic photo-induced strain field ($\boldsymbol{F}^p$) because of the *trans–cis* photoisomerization, which depends on the direction of the Mesogens ($\boldsymbol{N}_{\mathrm{M}}$) and the *cis* fraction n_c, and can be expressed as

$$\boldsymbol{F}^p = (1-\epsilon^p)\boldsymbol{N}_{\mathrm{M}}\otimes\boldsymbol{N}_M + (1+v^p\epsilon^p)(\boldsymbol{I}-\boldsymbol{N}_{\mathrm{M}}\otimes\boldsymbol{N}_M) \quad (S1)$$

where $\epsilon^p = \alpha^p n_c$ is the strain along the mesogen direction and v^p represents the effective Poisson's ratio. The evolution equation of the *cis* fraction n_c can be expressed as follows

$$\frac{\partial n_c}{\partial t} = -\frac{1}{\tau}n_c + \Gamma \mathcal{J} \quad (S2)$$

where τ represents the *cis* state lifetime, Γ is the adsorption constant, and $\mathcal{I}$ is the effective light intensity. For small n_c, the effective light intensity follows the Beer-Lambert law that exponentially decays from the surface to the bulk. In our model we simplify the spatial distribution of $\mathcal{I}$ along the thickness direction and divide the whole film into three layers, with the top and bottom layers 4 μm thick and response to light and the middle layer 12 μm thick and not response to light. However, the effective light intensity $\mathcal{I}$ in the top and bottom layers will be modified by the deformation, as illustrated in Fig. S33. When the surface rotates, its relative angle between the surface normal and the light angle will be changed and thus alter the light penetration. It has been shown that a projection of the global light intensity ($\mathcal{I}_0$) on to the current surface normal direction ($\boldsymbol{n}$) is good enough to capture this nonlinear photomechanical coupling. Thanks to the simplification of the single layer model, we can use the 3D rotational matrix ($\boldsymbol{R}$) in each layer to track the surface normal ($\boldsymbol{n} = \boldsymbol{R} \cdot \boldsymbol{n}_0, \boldsymbol{n}_0$ is the initial surface normal vector) and update the $\mathcal{I}$, such that

$$\mathcal{I} = \begin{cases} \mathcal{I}_0 |\boldsymbol{m} \cdot \boldsymbol{R} \cdot \boldsymbol{n}_0|, & \boldsymbol{m} \cdot \boldsymbol{R} \cdot \boldsymbol{n}_0 < 0 \\ 0, & \boldsymbol{m} \cdot \boldsymbol{R} \cdot \boldsymbol{n}_0 > 0 \end{cases} \tag{S3}$$

If $\boldsymbol{m} \cdot \boldsymbol{R} \cdot \boldsymbol{n}_0 > 0$, the surface is not directly facing the light, leading to a self-shadowing effect. This model can be seen as a generalization to 3D from the previous reduced models for 1D beams and 2D shells. We then integrate the active photogenerated strain into a hyper-elastic model through a multiplicative decomposition of the total deformation gradient tensor $\boldsymbol{F}$, such that

$$\boldsymbol{F} = \boldsymbol{F}^e \boldsymbol{F}^p \tag{S4}$$

where $F_{ij} = \frac{\partial x_i}{\partial X_j}, i, j = 1,2,3$, and $\boldsymbol{X}$ and $\boldsymbol{x}$ are the configurations before and after deformation, respectively, $\boldsymbol{F}^p$ is the photo part and $\boldsymbol{F}^e$ is the elastic part of $\boldsymbol{F}$. More specifically, we adopt the neo-Hookean model, whose strain energy density can be expressed in terms of the elastic component $\boldsymbol{F}^e$

$$U = \frac{1}{2}\mu\left(J^{e^{-2/3}} I_1^e - 3\right) + \frac{K}{2}\left(lnJ^e\right)^2 \tag{S5}$$

where μ is the shear modulus, K is the bulk modulus, $I_1^e = \mathrm{tr}(\boldsymbol{F}^{e^T}\boldsymbol{F}^e)$ is the first invariant of the right Cauchy-Green deformation tensor associated with $\boldsymbol{F}^e$ and $J^e = \det(\boldsymbol{F}^e)$. We implement the model into ABAQUS through the user defined material (UMAT) subroutine, where the *cis* fraction n_c is stored as a state variable in each material point and updated through a middle point time integration of the Eq. S2. It is noted that we do not intend to capture the detailed and complicated photo-thermo-mechanical coupling of the Azo-LCP but aim to predict the deformed shapes and snap through configurations of the LCP jumpers. Even in the case of this simplified model, we still have a large set of parameters to calibrate with experiments, which will be obtained from the mechanical measurements and the matching with experimental jumping behavior of uniform sheets. We focused on the Azo-LCP-5 and Azo-LCP-60 materials as well as their hybrid structures. The material properties used in the simulations are summarized in Table S5. Since we only use elastic model, we find the mass proportional Rayleigh damping coefficient (α_R) plays an important role in determining the jump height and tune it to closely match the experiments.

The simulations are carried out with ABAQUS/implicit (2022) using the general-purpose linear brick element with reduced integration (C3D8R). First, the Azo-LCP jumper is placed on a rigid substrate through a frictional contact with a friction coefficient of 0.5. It involves two steps with different sides exposed to the light, denoted as curling and flip-jumping step, respectively. The curling step is for 2 s. It should be noted that this period is shorter than the experiment because the temperature change in the experiment will make the Azo-LCP reach a static bending state longer. The flip-jumping step is about 1 s or until the jump finishes. In all the simulations, we assume the global light intensity is 0.3 W cm^{-2}.

Table S1. Summary of thermomechanical properties of Azo-LCP with uniform crosslinking density.

Sample	Curing time (min)	T_g (°C)	T_{high} (K)	E'_{high} (MPa)	v_e (mol m^{-3})
Azo-LCP-5	5	69.3 (±2.5)	392.3 (±2.5)	33.1 (±5.1)	3,382 (±499)
Azo-LCP-10	10	74.1 (±1.5)	397.1 (±1.5)	39.4 (±3.0)	4,178 (±285)
Azo-LCP-30	30	81.8 (±2.1)	404.8 (±2.1)	55.1 (±6.6)	5,456 (±634)
Azo-LCP-60	60	83.0 (±1.8)	406.0 (±1.8)	74.8 (±6.7)	7,388 (±634)

Table S2. Summary of mechanical properties of Azo-LCPs with different curing times.

Sample	Modulus (MPa)	Tensile strength (MPa)	Elongation at break (%)
Azo-LCP-5	6.82	15.02	6.75
Azo-LCP-10	7.49	15.93	4.95
Azo-LCP-30	13.57	16.21	2.72
Azo-LCP-60	18.89	16.43	1.34

Table S3. Jumping capability of various jumping actuators.

Ref.	Body mass (g)	Body length (cm)	Take-off velocity (BL s^{-2})	Height (BL)	Distance (BL)	Operating system
**This work**	**0.0002**	**0.2**	**585**	**24.5**	**12.5**	**Photomechanical coupling**
9	0.0002	0.2	880	15.5	N/A	Photomechanical coupling
44	0.001	1	62	10	N/A	Photomechanical coupling
33	0.0097	1.2	81	1.5	2.96	Photothermal heating
34	0.0022	1.3	615.4	0.7	0.5	Photothermal heating
43	0.00018	0.2	140.0	5.0	N/A	Photothermal heating
36	0.02804	0.25	12.0	12.0	0.4	Photothermal heating
32	0.672	1.3	123.1	6.2	8	Photothermal heating
45	0.0003	1.7	14.7	0.5	N/A	Photothermal heating
25	0.8	8	18.75	81.6	4.5	Magnetic field
26	0.0019	0.36	6.9	1.55	2.25	Magnetic field
27	10	5.8	24.8	2.4	4	Joule heating
28	0.324	2.94	5.8	20	5.5	Combustion
29	510	15	17.3	4.0	2	Combustion
30	964.6	11.8	9.0	6.4	0.5	Combustion
31	2100	18	6.7	0.5	2.8	Combustion

Table S4. Jumping capability of centimeter-scale living species.

Ref.	Animal	Body mass (mg)	Body length (cm)	Take-off velocity (BL s^{-2})	Height (BL)
39	Flea (*Archaeopsyllus erinacei*)	0.7	0.18	722.2	55.6
40	Spring tail	1.2	0.15	866.7	44
41	Locust (*L. migratoria*)	0.147	5.79	32.3	7.8
42	Froghopper (*Philaenus*)	12.3	0.61	770.5	115

Table S5. Material properties of Azo-LCP-5 and Azo-LCP-60 used for simulation.

	$\boldsymbol{\mu}$ (MPa)	$\boldsymbol{K}$ (MPa)	$\boldsymbol{\rho}$ (g cm^{-3})	$\boldsymbol{\alpha_R}$ (s^{-1})	$\boldsymbol{\tau}$ (s)	$\boldsymbol{\Gamma}$ (m^2 J^{-1})	$\boldsymbol{\alpha^P}$	$\boldsymbol{\nu^P}$
Azo-LCP-5	14.3	57.1	1.15	20	0.4	10^{-4}	0.72	0.5
Azo-LCP-60	28.6	114.2	1.3	40	0.6	0.6×10^{-4}	0.6	0.5

Movie S1.
Photomechanical jumps of Azo-LCPs with different crosslinking densities.
Movie S2.
Temperature effect on the photomechanical jump of Azo-LCP-5.
Movie S3.
Topological transition of uniformly crosslinked Azo-LCPs and asymmetrically patterned Azo-LCP.
Movie S4.
Directional jump of corner-rigid Azo-LCP.
Movie S5.
Directional take-off of corner-rigid Azo-LCP via tilted energy release.
Movie S6.
Efficient vertical jumps of center-rigid Azo-LCPs.
Movie S7.
Dual-mode Azo-LCP jumper: Blocking force-assisted snap-through and jump (Mode I).
Movie S8.
Dual-mode Azo-LCP jumper: Asymmetric, sequential snap-through and jump (Mode II).
Movie S9.
Sequential and consecutive dual-mode photomechanical jumps.

REFERENCES AND NOTES

1. M. L. Smith, G. M. Yanega, A. Ruina, Elastic instability model of rapid beak closure in hummingbirds. *J. Theor. Biol.* **282**, 41–51 (2011).

2. Y. Wang, Q. Wang, M. Liu, Y. Qin, L. Cheng, O. Bolmin, M. Alleyne, A. Wissa, R. H. Baughman, D. Vella, S. Tawfick, Insect-scale jumping robots enabled by a dynamic buckling cascade. *Proc. Natl. Acad. Sci. U.S.A.* **120**, e2210651120 (2023).

3. Y. Forterre, J. M. Skotheim, J. Dumals, L. Mahadevan, How the Venus flytrap snaps. *Nature* **433**, 421–425 (2005).

4. Y. Kim, J. van den Berg, A. J. Crosby, Autonomous snapping and jumping polymer gels. *Nat. Mater.* **20**, 1695–1701 (2021).

5. Y. Luo, D. K. Patel, Z. Li, Y. Hu, H. Luo, L. Yao, C. Majidi, Intrinsically multistable soft actuator driven by mixed-mode snap-through instabilities. *Adv. Sci.* **11**, e2307391 (2024).

6. B. Gorissen, D. Melancon, N. Vasios, M. Torbati, K. Bertoldi, Inflatable soft jumper inspired by shell snapping. *Sci. Robot.* **5**, eabb1967 (2020).

7. T. S. Hebner, K. Korner, C. N. Bowman, K. Bhattacharya, T. J. White, Leaping liquid crystal elastomers. *Sci. Adv.* **9**, eade1320 (2023).

8. Y. Yang, Y. Wang, Snapping for 4D-printed insect-scale metal-jumper. *Adv. Sci.* **11**, 2307088 (2024).

9. J. Jeon, J. C. Choi, H. Lee, W. Cho, K. Lee, J. G. Kim, J. W. Lee, K. Il Joo, M. Cho, H. R. Kim, J. J. Wie, Continuous and programmable photomechanical jumping of polymer monoliths. *Mater. Today* **49**, 97–106 (2021).

10. A. D. Shaw, S. A. Neild, D. J. Wagg, P. M. Weaver, A. Carrella, A nonlinear spring mechanism incorporating a bistable composite plate for vibration isolation. *J. Sound Vib.* **332**, 6265–6275 (2013).

11. M. Gomez, D. E. Moulton, D. Vella, Critical slowing down in purely elastic ‘snap-through’ instabilities. *Nat. Phys.* **13**, 142–145 (2017).

12. J. J. Wie, M. R. Shankar, T. J. White, Photomotility of polymers. *Nat. Commun.* **7**, 13260 (2016).

13. W. Cho, J. Jeon, W. Eom, J. G. Lee, D. G. Kim, Y. S. Kim, T. H. Han, J. J. Wie, Photo-triggered shape reconfiguration in stretchable reduced graphene oxide-patterned azobenzene-functionalized liquid crystalline polymer networks. *Adv. Funct. Mater.* **31**, 2102106 (2021).

14. W. Feng, Q. He, L. Zhang, Embedded physical intelligence in liquid crystalline polymer actuators and robots. *Adv. Mater.* **37**, 2312313 (2024).

15. J. G. Kim, J. Jeon, R. Sivakumar, J. Lee, Y. H. Kim, M. Cho, J. H. Youk, J. J. Wie, Light-fueled climbing of monolithic torsional soft robots via molecular engineering. *Adv. Intell. Syst.* **4**, 2100148 (2022).

16. J.-C. Choi, J. Jeon, J.-W. Lee, A. Nauman, J. G. Lee, W. Cho, C. Lee, Y.-M. Cho, J. J. Wie, H.-R. Kim, Steerable and agile light-fueled rolling locomotors by curvature-engineered torsional torque. *Adv. Sci.* **10**, 2304715 (2023).

17. C. Ma, Y. Zhang, S. Jiao, M. Liu, Snap-through of graphene nanowrinkles under out-of-plane compression. *Nanotechnology* **34**, 015705 (2023).

18. R. M. Springman, J. L. Bassani, Snap transitions in adhesion. *J. Mech. Phys. Solids* **56**, 2358–2380 (2008).

19. M. Ravi Shankar, M. L. Smith, V. P. Tondiglia, K. M. Lee, M. E. McConney, D. H. Wang, L. S. Tan, T. J. White, Contactless, photoinitiated snap-through in azobenzene-functionalized polymers. *Proc. Natl. Acad. Sci. U.S.A.* **110**, 18792–18797 (2013).

20. K. Yang, S. Won, J. E. Park, J. Jeon, J. J. Wie, Magnetic swarm intelligence of mass-produced, programmable microrobot assemblies for versatile task execution. *Device* **3**, 100626 (2025).

21. H. Moon, J. G. Lee, W. Cho, J. Jeon, J. E. Park, J. J. Wie, Structure-property-actuation relationships of shape-fixable magnetic vitrimer micropillar arrays. *Sens. Actuators B Chem.* **402**, 135092 (2024).

22. J. Jeon, H. Moon, J. Park, S. Won, J. E. Park, Z. Ku, J. O. Kim, J. J. Wie, Collective and rapid high amplitude magnetic oscillation of anisotropic micropillar arrays. *ACS Nano* **19**, 9946–9957 (2025).

23. P. J. Flory, Molecular theory of rubber elasticity. *Polym. J.* **20**, 1–12 (1985).

24. H. M. D. Bandara, S. C. Burdette, Photoisomerization in different classes of azobenzene. *Chem. Soc. Rev.* **41**, 1809–1825 (2012).

25. K. Korner, A. S. Kuenstler, R. C. Hayward, B. Audoly, K. Bhattacharya, A nonlinear beam model of photomotile structures. *Proc. Natl. Acad. Sci. U.S.A.* **117**, 9762–9770 (2020).

26. F. Dadgar-Rad, M. M. Mahjoub, M. Hossain, A hyperelastic beam model for the photo-induced response of nematic liquid crystal elastomers. *Extrem. Mech. Lett.* **72**, 102233 (2024).

27. D. Liu, D. J. Broer, Liquid crystal polymer networks: Preparation, properties, and applications of films with patterned molecular alignment. *Langmuir* **30**, 13499–13509 (2014).

28. D. J. Broer, R. G. Gossink, R. A. M. Hikmet, Oriented polymer networks obtained by photopolymerization of liquid-crystalline monomers. *Angew. Makromol. Chem.* **183**, 45–66 (1990).

29. J. Wang, D. Wang, A lightweight jumping robot with untethered actuation. *Lect. Notes Comput. Sci.* **14270**, 71–82 (2023).

30. W. Hu, G. Z. Lum, M. Mastrangeli, M. Sitti, Small-scale soft-bodied robot with multimodal locomotion. *Nature* **554**, 81–85 (2018).

31. Z. Zhakypov, K. Mori, K. Hosoda, J. Paik, Designing minimal and scalable insect-inspired multi-locomotion millirobots. *Nature* **571**, 381–386 (2019).

32. C. A. Aubin, R. H. Heisser, O. Peretz, J. Timko, J. Lo, E. F. Helbling, S. Sobhani, A. D. Gat, R. F. Shepherd, Powerful, soft combustion actuators for insect-scale robots. *Science* **381**, 1212–1217 (2023).

33. M. T. Tolley, R. F. Shepherd, M. Karpelson, N. W. Bartlett, K. C. Galloway, M. Wehner, R. Nunes, G. M. Whitesides, R. J. Wood, “An untethered jumping soft robot” in *2014 IEEE/RSJ International Conference on Intelligent Robots and Systems* (IEEE, 2014), pp. 561–566.

34. N. W. Bartlett, M. T. Tolley, J. T. B. Overvelde, J. C. Weaver, B. Mosadegh, K. Bertoldi, G. M. Whitesides, R. J. Wood, A 3D-printed, functionally graded soft robot powered by combustion. *Science* **349**, 161–165 (2015).

35. M. Loepfe, C. M. Schumacher, U. B. Lustenberger, W. J. Stark, An untethered, jumping roly-poly soft robot driven by combustion. *Soft Robot.* **2**, 33–41 (2015).

36. C. Ahn, X. Liang, S. Cai, Bioinspired design of light-powered crawling, squeezing, and jumping untethered soft robot. *Adv. Mater. Technol.* **4**, 1900185 (2019).

37. W. Cho, D. J. Kang, M. J. Hahm, J. Jeon, D. G. Kim, Y. S. Kim, T. H. Han, J. J. Wie, Multi-functional locomotion of collectively assembled shape-reconfigurable electronics. *Nano Energy* **118**, 108953 (2023).

38. H. Guo, A. Priimagi, H. Zeng, Optically controlled latching and launching in soft actuators. *Adv. Funct. Mater.* **32**, 2108919 (2022).

39. J. Hu, Z. Nie, M. Wang, Z. Liu, S. Huang, H. Yang, Springtail-inspired light-driven soft jumping robots based on liquid crystal elastomers with monolithic three-leaf panel fold structure. *Angew. Chem. Int. Ed.* **62**, e202218227 (2023).

40. Y. Hu, J. Liu, L. Chang, L. Yang, A. Xu, K. Qi, P. Lu, G. Wu, W. Chen, Y. Wu, Electrically and sunlight-driven actuator with versatile biomimetic motions based on rolled carbon nanotube bilayer composite. *Adv. Funct. Mater.* **27**, 1704388 (2017).

41. G. P. Sutton, M. Burrows, Biomechanics of jumping in the flea. *J. Exp. Biol.* **214**, 836–847 (2011).

42. S. Sudo, T. Kainuma, T. Yano, A. Shirai, T. Hayase, Jumps of water springtail and morphology of the jumping organ. *J. Jpn. Soc. Exp. Mech.* **15**, s117–s124 (2015).

43. X. Mo, D. Romano, M. Miraglia, W. Ge, C. Stefanini, Effect of substrates' compliance on the jumping mechanism of locusta migratoria. *Front. Bioeng. Biotechnol.* **8**, 661 (2020).

44. M. Burrows, Jumping performance of froghopper insects. *J. Exp. Biol.* **209**, 4607–4621 (2006).

45. H. Arazoe, D. Miyajima, K. Akaike, F. Araoka, E. Sato, T. Hikima, M. Kawamoto, T. Aida, An autonomous actuator driven by fluctuations in ambient humidity. *Nat. Mater.* **15**, 1084–1089 (2016).

46. B. Lei, Z. Y. Wen, H. K. Wang, J. Gao, L. J. Chen, Bioinspired jumping soft actuators of the liquid crystal elastomer enabled by photo-mechanical coupling. *ACS Appl. Mater. Interfaces* **16**, 1596–1604 (2024).

47. H. Li, J. Wang, Ultrafast yet controllable dual-responsive all-carbon actuators for implementing unusual mechanical movements. *ACS Appl. Mater. Interfaces* **11**, 10218–10225 (2019).

48. D. Liu, D. J. Broer, New insights into photoactivated volume generation boost surface morphing in liquid crystal coatings. *Nat. Commun.* **6**, 8334 (2015).

49. T. J. White, D. J. Broer, Programmable and adaptive mechanics with liquid crystal polymer networks and elastomers. *Nat. Mater.* **14**, 1087–1098 (2015).

50. U. Hrozhyk, S. Serak, N. Tabiryan, T. J. White, T. J. Bunning, Bidirectional photoresponse of surface pretreated azobenzene liquid crystal polymer networks. *Opt. Express* **17**, 716–722 (2009).
